# Chromone Derivatives with α-Glucosidase Inhibitory Activity from the Marine Fungus *Penicillium thomii* Maire

**DOI:** 10.3390/molecules26175273

**Published:** 2021-08-31

**Authors:** Shouye Han, Yu Liu, Wan Liu, Fan Yang, Jia Zhang, Ruifeng Liu, Fenqin Zhao, Wei Xu, Zhongbin Cheng

**Affiliations:** 1 School of Pharmacy, Henan University, Kaifeng 475004, China; hanshouye123@163.com (S.H.); liuyu5230710@163.com (Y.L.); 18737806806@163.com (W.L.); Y18992588130@126.com (F.Y.); z919395@126.com (J.Z.); liurf12138@163.com (R.L.); 2 Key Laboratory of Marine Biogenetic Resources, Third Institute of Oceanography, Ministry of Natural Resources, Xiamen 361005, China

**Keywords:** chromone derivatives, structural elucidation, inhibitions on α-glucosidase

## Abstract

The fungal strain YPGA3 was isolated from the sediments of the Yap Trench and identified as *Penicillium thomii*. Eight new chromone derivatives, named penithochromones M−T (**1**–**8**), along with two known analogues, **9** and **10,** were isolated from the strain. The structures were established by detailed analyses of the spectroscopic data. The absolute configuration of the only chiral center in compound **1** was tentatively determined by comparing the experimental and the calculated specific rotations. Compounds **7** and **8** represent the first examples of chromone derivatives featuring a 5,7-dioxygenated chromone moiety with a 9-carbon side chain. Bioassay study revealed that compounds **6**–**10** exhibited remarkable inhibition against α-glucosidase with IC_50_ values ranging from 268 to 1017 μM, which are more active than the positive control acarbose (1.3 mmol).

## 1. Introduction

Chromone is a group of oxygen-bearing heterocyclic molecules featured by a benzoannelated γ-pyrone ring. Natural products containing a chromone moiety are widespread in nature and display various biological activities, such as antioxidant, antiviral, and anti-inflammatory activities [1]. In recent years, the chemical study of marine fungi has led to the discovery of numerous new or bioactive compounds, contributing greatly to natural products chemistry [2,3,4,5,6]. Among these metabolites, some chromone derivatives exhibit significant activity, the following are some examples. Pestalotiochromone A, isolated from the fungus *Pestalotiopsis neglecta*, exhibited potent binding affinities with liver X receptor α with the dissociation equilibrium constant value 6.2 μM [7]. Pestalotiopsones B and F isolated from *Diaporthe* sp. displayed potential antiviral activities against three influenza A virus subtypes with IC_50_ values ranging from 2.56 to 6.76 μM [8]. The rare dihydrothiophene-condensed chromones oxalicumones A, B, D, and E from *Penicillium oxalicum* showed notable cytotoxicity against several carcinoma cell lines with IC_50_ values below 10 μM [9]. The active chromone derivatives may provide a structural basis for the research and development of related new drugs.

**Figure 1:**
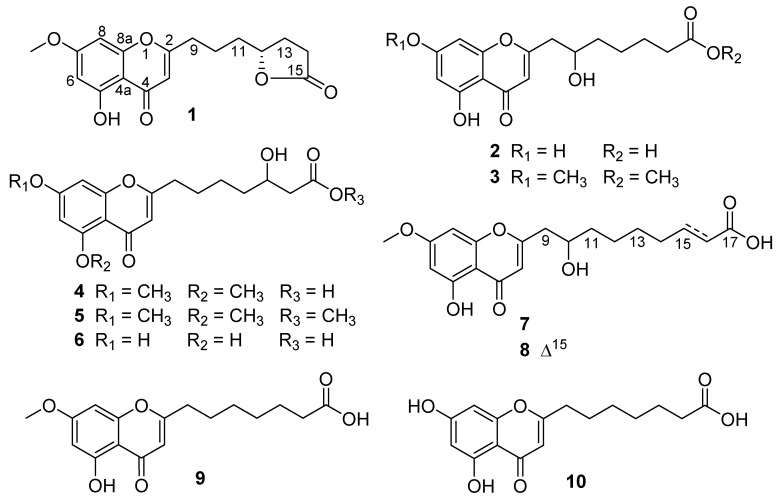
Structures of compounds **1**–**10** from *Penicillium thomii* YPGA3.

In our efforts to search for bioactive molecules from deep-sea fungus [10,11,12,13,14], the fungal strain *Penicillium thomii* YPGA3 isolated from the sediments at a depth of 4500 m in the Yap Trench was screened out for chemical investigation, and the ^1^H NMR spectrum and HPLC-DAD fingerprint of the EtOAc extract of this strain presented information that suggested the presence of chromone derivatives. As a result, eight new chromone derivatives, namely penithochromones M−T (**1**–**8**), along with two known analogues, **9** and **10,** were obtained by ^1^H NMR-guided isolation (Figure 1). All compounds were evaluated for their inhibitions against α-glucosidase and the antioxidant capacities. Herein, we report the structural elucidation of the new chromone derivatives and the bioactivities.

## 2. Results

**Figure 2:**
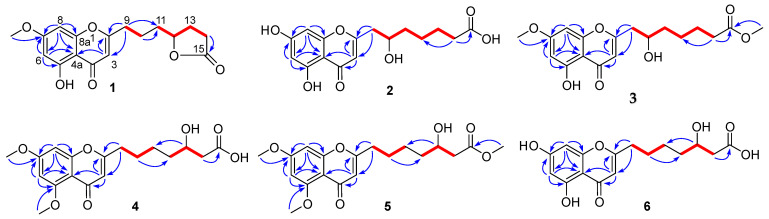
^1^H-^1^H COSY (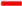) and HMBC (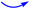) correlations of **1**–**6**.

Penithochromone M (**1**) was obtained as a light-yellow oil. The HRESIMS data gave a molecular formula of C_17_H_18_O_6_, requiring nine indices of hydrogen deficiency. The ^1^H NMR spectrum of **1** showed resonances for two meta-coupled aromatic protons at δ_H_ 6.36 (1H, d, *J* = 1.9 Hz, H-6) and 6.61 (1H, d, *J* = 1.9 Hz, H-8), a singlet for an olefinic resonance at δ_H_ 6.25 (1H, s, H-3), a methoxy (δ_H_ 3.84), an oxygenated methine (δ_H_ 4.52), and ten aliphatic protons (Table 1 and Figure S1). The ^13^C NMR and HSQC spectra (Figures S2 and S3) displayed 17 carbon resonances including two carbonyl groups including a ketone carbon (δ_C_ 182.0, 177.1), eight aromatic or olefinic carbons, a methoxy (δ_C_ 56.1), an oxygenated methine (δ_C_ 79.9), and five methylenes (δ_C_ 34.0, 32.9, 28.3, 27.3, 22.3). The aforementioned data indicated a chromone derivative, structurally similar to the co-isolated compound penithochromone C (**9**) [12]. The chromone moiety was assigned to be the same as that of penithochromone C by HMBC correlations from the protons H-6 (δ_H_ 6.36), H-8 (δ_H_ 6.61), and H-3 (δ_H_ 6.25) to the aromatic or olefinic carbons in association with the correlation from the methoxy protons at δ_H_ 3.84 to the oxygenated aromatic carbon C-7 (δ_C_ 165.2) (Figure 2 and Figure S5). The rest resonances are attributed to a seven-carbon unit located at C-2 by analysis of 2D NMR data (Figure 2 and Figures S3–S6). The ^1^H-^1^H COSY spectrum displayed two spin systems formed by H_2_-9 (δ_H_ 2.69)/H_2_-10 (δ_H_ 1.77) and H_2_-11 (δ_H_ 1.69)/H-12 (δ_H_ 4.52)/H_2_-13 (δ_H_ 2.27, 1.80)/H_2_-14 (δ_H_ 2.49). They were connected by the HMBC correlations from H_2_-9 and H_2_-10 to C-11 (δ_C_ 34.0). The carbonyl carbon at δ_C_ 177.1 was adjacent to the methylene CH_2_-14 by HMBC correlation from H_2_-14 to C-15 (δ_C_ 177.1). As the chromone nucleus and the carbonyl carbon C-15 accounted for eight degrees of unsaturation, the remaining one required the presence of an additional ring in the seven-carbon unit. Taking the chemical shifts of H-12 and C-15 and the MS data into consideration, the methine carbon C-12 and the carbonyl carbon C-15 should be connected via an O-atom to form a five-membered lactone ring. HMBC correlations from H_2_-9 to C-2 and C-3 positioned the seven-carbon unit at C-2. Thus, the gross structure of **1** was determined as depicted. The absolute configuration of the only chiral center C-12 in **1** was tentatively determined by comparing the experimental and the calculated specific rotation. Theoretical specific rotations of the model molecules *S*/*R*-**1** were calculated at the b3lyp/6-31+g(d) level using methanol as solvent. The results (Table S1) were that the theoretical specific rotation of *R*-**1** (*S*-**1**: [α${]}_{D}^{20}$ − 175.60; *R*-**1**: [α${]}_{D}^{20}$ + 175.60) was nearly the same as the experimental value ([α${]}_{D}^{20}$ + 211). Thus, the absolute configuration of C-12 was tentatively assigned as *R*.

The molecular formula of penithochromone N (**2**) was determined to be C_16_H_18_O_7_ by the HRESIMS data. The ^1^H NMR spectrum showed the resonances for a 5,7-dihydroxychromone moiety (δ_H_ 6.10, 6.18, 6.33), an oxygenated proton (δ_H_ 4.0), and a series of aliphatic protons (Table 1 and Figure S7). The ^13^C NMR spectrum exhibited a total of 16 carbon resonances (Figure S8), including eight aromatic carbons for a benzene ring and a double bond, a carboxylic acid (δ_C_ 177.7), a ketone carbonyl (δ_C_ 183.9), five methylenes (δ_C_ 26.0, 26.3, 35.0, 38.1, 43.2), and an oxygenated methine (δ_C_ 70.0). The above-mentioned information was very similar to that of the co-isolated penithochromone A (**10**), with obvious distinction due to the presence of an oxygenated methine (δ_H_ 4.0; δ_C_ 70.0) and one less methylene [12]. As the molecular formula of **2** possessed one more O atom than that of penithochromone A, compound **2** was proposed to be hydroxylated derivative of penithochromone A. The hydroxyl group was positioned at C-10 by the COSY correlations from H_2_-9 (δ_H_ 2.81; 2.63) to H-10 (δ_H_ 4.0). Thus, the gross structure of **2** was determined as shown and was secured by 2D NMR analyses (Figure 2 and Figures S9–S12).

Penithochromone O (**3**) had the molecular formula C_18_H_22_O_7_. The NMR data of **3** were quite similar to those of **2** (Table 1 and Figures S13–S18). The structural differences were found to be the presences of two methoxy groups (δ_H_ 3.65, 3.87; δ_C_ 52.0, 56.5). The one at δ_H_ 3.87 was positioned at C-7 by HMBC correlation from the protons at δ_H_ 3.87 to C-7 (δ_C_ 167.4) (Figure 2), while the other one was positioned at C-15 by the cross peak δ_H_ 3.65/δ_C_ 176.0 in the HMBC spectrum. The structure of **3** was thus determined as depicted.

Penithochromone P (**4**) was isolated as a light-yellow oil. The HRESIMS data gave a molecular formula of C_18_H_22_O_7_, which was the same as that of **3**, suggesting that they were isomers. The NMR spectra showed similar structural features as those of **3** (Table 1 and Table 2). Based on analyses of the 1D and 2D NMR data (Figure 2 and Figures S19–S24), the differences between **4** and **3** were owing to the positions of the hydroxyl in the side chain and the two methoxy groups. With HMBC correlations from the protons at δ_H_ 3.89 to C-5 (δ_C_ 162.1) and the protons at 3.90 to C-7 (δ_C_ 166.3), the two methoxy groups were located at C-5 and C-7, respectively. The hydroxyl group in the side chain was placed at C-13 (δ_C_ 69.3) by the spin system H_2_-9/H_2_-10/H_2_-11 /H_2_-12/H-13/H_2_-14 observed in the COSY spectrum.

The NMR data of penithochromone Q (**5**) were nearly the same as those of **4** (Table 2 and Figures S25–S30). The only difference between **4** and **5** was found by the presence of an additional methoxy group (δ_H_ 3.67, δ_C_ 52.0) in **5**, which was placed at C-15 by the HMBC correlation from the methoxy protons at δ_H_ 3.67 to the carbonyl carbon at δ_C_ 174.0. The structure of **5** was further confirmed by detailed analyses of the 2D NMR data (Figure 2).

The NMR data of penithochromone R (**6**) indicated that **6** was structurally similar to that of compound **4** (Table 2 and Figures S31–S36). The distinctions were attributed to the absence of the two aromatic methoxy groups, indicating that **6** was the corresponding demethylated derivative. The deduction was corroborated by 2D NMR analyses (Figure 2).

**Figure 3:**
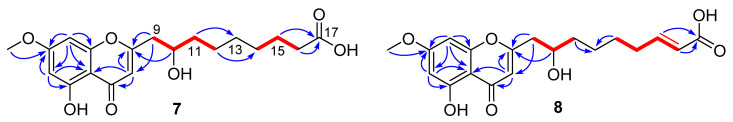
^1^H-^1^H COSY (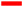) and HMBC (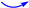) correlations of **7** and **8**.

Penithochromone S (**7**), a light-yellow oil, had the molecular formula C_19_H_24_O_7_ as provided by HRESIMS data. The NMR data of **7** indicated the structure consisted of a chromone moiety and a side chain (Table 3 and Figures S37–S41). The chromone moiety was determined to be the same as that of **1** by 2D NMR analyses (Figure 3). As for the side chain, two spin systems made up by H_2_-9/H-10/H_2_-11/H_2_-12 and H_2_-14/H_2_-15/H_2_-16 can be deduced based on the COSY peaks, they were connected by HMBC correlations from H_2_-11 (δ_H_ 1.55) to C-13 (δ_C_ 30.3) and H_2_-12 (δ_H_ 1.41) to C-14 (δ_C_ 30.2). Additional HMBC correlations from H_2_-15 (δ_H_ 1.62) and H_2_-16 (δ_H_ 2.28) to the carboxylic acid carbon C-17 (δ_C_ 177.9) finally established a 9-carbon side chain, which was positioned at C-2 (δ_C_ 170.4) by HMBC correlations from H_2_-9 (δ_H_ 2.82, 2.65) to C-2 and C-3 (δ_C_ 110.4) and H-10 (δ_H_ 4.0) to C-2.

Penithochromone T (**8**) had the molecular formula C_19_H_22_O_7_. With similar 1D and 2D NMR data to **7** (Table 3 and Figures S42–S46), **8** was also determined to be a chromone derivative bearing a nine-carbon side chain. The distinctions can be found to be the presence of one *trans*-double bond [δ_H_ 6.95 (1H, dd, *J* = 15.7, 7.0 Hz), 5.81 (1H, d, *J* = 15.7 Hz); δ_C_ 150.6, 123.0] and the absence of two methylenes. The double bond was positioned at C-15 and C-16 by HMBC correlations from H-15 (δ_H_ 6.95) and H-16 (δ_H_ 5.81) to the carboxylic acid carbon C-17 (δ_C_ 170.5). The structure of **8** was supported by detailed analyses of the 2D NMR data (Figure 2).

The structures of compounds **1**–**8** featured a 5,7-dioxygenated chromone moiety with a 7 or 9-carbon alkyl acid or alkyl ester side chain substituted at C-2. Previously reported analogs including penithochromones A−L [12], 4-(5,7-dimethoxy-4-oxo-4H-chromen-2-yl)butanoic acid [15], and 3-(5,7-dimethoxy-4-oxo-4H-chromen-2-yl)propanoic acid [15] possessed similar alkyl acid or alkyl ester side chain with lengths of 3, 4, 5, or 7 carbon atoms (Figure S55). The side chains of new compounds in the current study are hydroxylated or further dehydrated to form a lactone moiety comparing with those of reported analogues, and compounds **7** and **8** are the first cases of chromone derivatives featuring a 5,7-dioxygenated chromone moiety with a 9-carbon side chain.

α-Glucosidase inhibitors can prevent the digestion of carbohydrates and decrease the effect of carbohydrates on blood glucose, and are an effective therapy for patients with type 2 diabetes mellitus. As the known analog 4-(5,7-dimethoxy-4-oxo-4H-chromen-2-yl)butanoic acid exhibited moderate inhibitory activity on α-glucosidase [15], and some molecules containing a chromone moiety in the literature showed notable inhibitions against α-glucosidase [16], compounds **1**–**10** were screened for their α-glucosidase inhibitory effects at an initial concentration of 667 μM. Those with inhibitions more than 25% were further evaluated to determine the IC_50_ values (Table 4). The results showed that compounds **9** and **10** displayed significant inhibitory effects with IC_50_ values of 688 and 268 μM respectively, being much more effective than the positive control acarbose (1.33 mM). While compounds **7** and **8** exhibited comparable effects as acarbose with IC_50_ values of 917 and 1017 μM, respectively. Based on analysis of the structures and activities of **1**–**10**, the introduction of hydroxyl group in the side chain may decrease the inhibitory effects, as compound **9** was much more active than its hydroxylated derivatives **2** and **6**.

As compounds containing a phenolic moiety usually display effective antioxidant activity and could neutralize free radicals, thus preventing them from causing damage [17], compounds **1**–**10** were further evaluated for their antioxidant activity at the initial concentration of 1 mM. The results showed that all tested compounds exhibited very weak inhibitions less than 50% (Table 4).

## 3. Materials and Methods

### 3.1. General Experimental Procedure

Specific rotations were measured by an SGW-1 automatic polarimeter (Shanghai Jing Ke Industrial Co., Ltd., Shanghai, China). The NMR spectra were recorded on a Bruker Avance III HD-400 NMR spectrometer. HRESIMS spectra were obtained on a Waters Xevo G2 Q-TOF spectrometer fitted with an ESI source (Bruker Corporation, Karlsruhe, Germany). Semi-preparative high-performance liquid chromatography (HPLC) was undertaken on a Shimadzu LC-6AD pump (Shimadzu Co., Kyoto, Japan) using a UV detector, and a YMC-Pack ODS-A HPLC column (semipreparative, 250 × 10 mm, S-5 μm, 12 nm, YMC Co., Ltd., Kyoto, Japan) was used for separation.

### 3.2. Fungal Strain and Identification

The fungus YPGA3 was isolated from the deep-sea sediments at a depth of 4500 m in the Yap Trench (West Pacific Ocean). The strain was identified as *Penicillium thomii* based on microscopic examination and by internal transcribed spacer (ITS) sequencing. The ITS sequence has been deposited in GenBank (http://www.ncbi.nlm.nih.gov, accessed on 18 July 2019) with the accession number MG835903. The strain YPGA3 (MCCC 3A01052) was deposited at the Marine at the Marine Culture Collection of China.

### 3.3. Fermentation, Extraction, and Isolation

The fermentation was conducted in 30 fernbach flasks (500 mL), each containing 70 g of rice. Artificial seawater (90 mL) was added to each flask, and the contents were soaked for 3 h before autoclaving at 15 psi for 30 min. After cooling to room temperature (rt), each flask was inoculated with 3.0 mL of the spore inoculum and incubated at rt for 30 days. The fermented material was extracted with EtOAc (3 × 4000 mL). The extracting solution was evaporated to dryness under reduced pressure to give an extract (3.1 g), which was subjected to a middle chromatogram isolated gel (MCI) with MeOH/H_2_O (10:90→100:0) as eluent to obtain 10 fractions (F1 to F10). Fraction F1 was fractionated over C18 reversed-phase (RP-18) silica gel with MeOH/H_2_O (10:90→90:10) to afford five subfractions (SF1a–SF1e). SF1b was separated on a semipreparative reversed-phase (RP)-HPLC column using MeCN/H_2_O = 28:72 (3 mL/min) to give **2** (t_R_ = 30.2 min, 2.4 mg) and **6** (t_R_ = 30.6 min, 3.8 mg. SF1d was separated on a RP-18 silica gel (MeOH/H_2_O, 30:70→90:10) to give six fractions (SF1d1–SF1d6). SF1d5 was purified by RP-HPLC using MeCN/H_2_O = 35:65 (3 mL/min) to afford **4** (t_R_ = 18.5 min, 2.3 mg). Fraction F3 was subjected to RP-18 silica gel eluted with MeOH/H_2_O (30:70→90:10) to collect 14 subfractions (SF3a–SF3n). SF3d was separated on HPLC using MeCN/H_2_O = 54:46 (3 mL/min) as eluent to afford **1** (t_R_ = 24.5 min, 1.9 mg) and **5** (t_R_ = 33.1 min, 3.6 mg). SF3f was purified on HPLC with MeCN/H_2_O = 51.5:48.5 (3 mL/min) to give **8** (t_R_ = 22.6 min, 1.8 mg), and **7** (t_R_ = 26.9 min, 3.2 mg), and **3** (t_R_ = 42.6 min, 2.0 mg).

Penithochromone M (**1**): light yellow oil; [α${]}_{D}^{20}$ + 211 (c 0.04, MeOH); UV (MeOH) λ_max_ 248, 292 nm; HRESIMS *m*/*z* 317.1021 [M−H]^−^ (calcd for C_17_H_17_O_6_^−^, 317.1031), HRESIMS *m*/*z* 319.1172 [M + H]^+^ (calcd for C_17_H_19_O_6_^+^, 319.1176) (Figure S47).

Penithochromone N (**2**): light yellow oil; [α${]}_{D}^{20}$ + 147 (c 0.24, MeOH); UV (MeOH) λ_max_ 248, 295 nm; HRESIMS *m*/*z* 321.0970 [M−H]^−^ (calcd for C_16_H_17_O_7_^−^, 321.0980), HRESIMS *m*/*z* 345.0933 [M + Na]^+^ (calcd for C_16_H_18_O_7_Na^+^, 345.0945) (Figure S48).

Penithochromone O (**3**): light yellow oil; [α${]}_{D}^{20}$ + 300 (c 0.04, MeOH); UV (MeOH) λ_max_ 248, 293 nm; HRESIMS *m*/*z* 349.1277 [M−H]^−^ (calcd for C_18_H_21_O_7_^−^, 349.1293), HRESIMS *m*/*z* 351.1425 [M + H]^+^ (calcd for C_18_H_23_O_7_^+^, 351.1438) (Figure S49).

Penithochromone P (**4**): light yellow oil; [α${]}_{D}^{20}$ + 216 (c 0.03, MeOH); UV (MeOH) λ_max_ 232, 287 nm; HRESIMS *m*/*z* 349.1271 [M−H]^−^ (calcd for C_18_H_21_O_7_^−^, 349.1293), HRESIMS *m*/*z* 351.1413 [M + H]^+^ (calcd for C_18_H_23_O_7_^+^, 351.1438), HRESIMS *m*/*z* 373.1222 [M + Na]^+^ (calcd for C_18_H_22_O_7_Na^+^, 373.1258) (Figure S50).

Penithochromone Q (**5**): light yellow oil; [α${]}_{D}^{20}$ + 173 (c 0.06, MeOH); UV (MeOH) λ_max_ 252, 291 nm; HRESIMS *m*/*z* 365.1580 [M + H]^+^ (calcd for C_19_H_25_O_7_^+^, 365.1595), HRESIMS *m*/*z* 387.1401 [M + Na]^+^ (calcd for C_19_H_24_O_7_Na^+^, 387.1414) (Figure S51).

Penithochromone R (**6**): light yellow oil; [α${]}_{D}^{20}$ + 153 (c 0.04, MeOH); UV (MeOH) λ_max_ 247, 294 nm; HRESIMS *m*/*z* 321.0973 [M−H]^−^ (calcd for C_16_H_17_O_7_^−^, 321.0980), HRESIMS *m*/*z* 345.0927 [M + Na]^+^ (calcd for C_16_H_18_O_7_Na^+^, 345.0945) (Figure S52).

Penithochromone S (**7**): light yellow oil; [α${]}_{D}^{20}$ + 138 (c 0.07, MeOH); UV (MeOH) λ_max_ 248, 293 nm; HRESIMS *m*/*z* 363.1442 [M−H]^−^ (calcd for C_19_H_23_O_7_^−^, 363.1449), HRESIMS *m*/*z* 387.1410 [M + Na]^+^ (calcd for C_19_H_24_O_7_Na^+^, 387.1414) (Figure S53).

Penithochromone T (**8**): light yellow oil; [α${]}_{D}^{20}$ + 240 (c 0.04, MeOH); UV (MeOH) λ_max_ 248, 292 nm; HRESIMS *m*/*z* 363.1450 [M + H]^+^ (calcd for C_19_H_23_O_7_^+^, 363.1438), HRESIMS *m*/*z* 385.1253 [M + Na]^+^ (calcd for C_19_H_22_O_7_Na^+^, 385.1258) (Figure S54).

### 3.4. α-Glucosidase Assay

The α-glucosidase inhibitory effect was performed as follows [18]. The 0.2 U of α-glucosidase from *Saccharomyes cerevisiae,* purchased from Sigma-Aldrich (St. Louis, MO, USA), was diluted to 0.067 M phosphate buffer consisting of Na_2_HPO_4_·12H_2_O and KH_2_PO_4_ (pH 6.8). The assay was conducted in a 60 μL reaction system containing 20 μL of diluted enzyme solution and 20 μL of DMSO or sample (dissolved in DMSO). After 10 min of incubation in the 96-well plates at 37 °C, 20 μL of 4 mM PNPG (4-nitrophenyl-α-*D*-glucopyranoside, Aladdin, Shanghai, China) was added as substrate to start the enzymatic reaction. The plate was incubated for an additional 20 min at 37 °C, and the reaction was quenched by adding 60 μL of 0.2 M Na_2_CO_3_. The final concentrations of tested compounds were between 0.2 and 2 mM. The optical density (OD) was measured at an absorbance wavelength of 405 nm using a Microplate Reader (Tecan, Switzerland). All assays were performed in three replicates, and acarbose (Aladdin, Shanghai, China) was used as the positive control. Percent inhibition was calculated by the following equation: Inhibition (%) = ((Absorbance of control − Absorbance of test)/Absorbance of control)); IC_50_ concentrations are calculated using concentration vs. percent inhibition values.

### 3.5. Antioxidant Activity

The DPPH scavenging was assayed following the procedures in our recent published paper [19]. The DPPH radical scavenging test was performed in 96-well microplates. Then, 20 μL of samples at a concentration of 10 mM in MeOH were added to 180 μL (150 μmol/L) DPPH solution. After 30 min of light avoidance, absorbance at 517 nm using a Microplate Reader (Tecan, Switzerland) was measured and the percentage of activity was calculated. All assays were performed in three replicates, and vitamin C was used as a positive control. Percent inhibition was calculated by the following equation: Inhibition (%) = ((Absorbance of control − Absorbance of test)/Absorbance of control)); IC_50_ concentrations are calculated using concentration vs. percent inhibition values.

### 3.6. Computational Details

Conformational analyses were carried out via random searching in the Sybyl-X 2.0 using the MMFF94S force field with an energy cutoff of 2.0 kcal/mol [20]. The results showed three lowest energy conformers for 12*S*-**1**. The conformers were re-optimized using DFT at the b3lyp/6-31+g(d,p) level in methanol using the solvation model based on density (smd) by the GAUSSIAN 09 program [21]. The specific rotations for each conformer were calculated using the TDDFT methodology at the b3lyp/6-31+g(d) level in methanol (Table S1). The specific rotations obtained for the conformers were averaged according to the Boltzmann distribution theory and their relative Gibbs free energy (ΔG) to get the final specific rotations (Table S1). The specific rotations of the corresponding enantiomers were determined by inversing the values of the calculated ones. By comparing the experiment data with the calculated data, the absolute configuration of the only chiral center in **1** was resolved to be *R*.

## 4. Conclusions

In the current study, chemical research of the deep-sea fungus *Penicillium thomii* YPGA3 resulted in the isolation of eight new chromone derivatives named penithochromones M−T (**1**–**8**), together with penithochromones C (**9**) and A (**10**). The structures were established by analyses of the NMR and HRESI data. Compounds **7** and **8** represent the first examples of chromone derivatives featuring a 5,7-dioxygenated chromone moiety with a 9-carbon side chain. Penithochromones A and C displayed conspicuous inhibition on α-glucosidase with IC_50_ values of 268 and 688 μM respectively, being much more effective than the positive control acarbose (1.3 mmol). Our study enriched the family of chromone derivatives and may provide ideas for the development of α-glucosidase inhibitors.

## Figures and Tables

**Table 1:** ^1^H NMR Data of Compounds **1**–**3** (δ in ppm, *J* in Hz).

Position	1 ^a^		2 ^b^		3 ^b^	
	δ_H_	δ_C_	δ_H_	δ_C_	δ_H_	δ_C_
2		170.6		170.0		170.4
3	6.25, s	107.8	6.10, s	110.1	6.15, s	110.4
4		182.0		183.9		184.0
4a		104.6		105.3		106.2
5		161.3		163.2		163.0
6	6.36, d (1.9)	98.0	6.18, d (1.9)	100.0	6.33, d (2.0)	99.2
7		165.2		165.9		167.4
8	6.61, d (1.9)	92.5	6.33, d (1.9)	95.0	6.54, d (2.0)	93.5
8a		157.8		159.9		159.8
9	2.69, t (6.5)	32.9	2.81, dd (14.5, 3.9) 2.63, dd (14.5, 8.8)	43.2	2.82, dd (14.3, 3.9) 2.66, dd (14.3, 8.7)	43.2
10	1.77, m	22.3	4.0, m	70.0	4.0, m	69.9
11	1.69, m	34.0	1.57, m	38.1	1.56, m	38.0
12	4.52, m	79.9	1.45, m 1.56, m	26.3	1.43, m 1.54, m	26.2
13	2.27, m 1.80, m	27.3	1.65, m	26.0	1.65, m	25.9
14	2.49, m	28.3	2.32, t (7.2)	35.0	2.35, t (7.3)	34.7
15		177.1		177.7		176.0
5-OCH_3_						
7-OCH_3_	3.84, s	56.1			3.87, s	56.5
15-OCH_3_					3.65, s	52.0

^a^ DMSO-*d*_6_, ^b^ in Methanol-*d*_4_.

**Table 2:** ^1^H NMR Data of Compounds **4**–**6** in Methanol-*d*_4_ (δ in ppm, *J* in Hz).

Position	4		5		6	
	δ_H_	δ_C_	δ_H_	δ_C_	δ_H_	δ_C_
2		169.7		169.6		172.4
3	6.04, s	111.3	6.05, s	111.3	6.05, s	108.4
4		180.1		180.1		184.0
4a		109.1		109.2		105.2
5		162.1		162.1		163.2
6	6.49, d (2.1)	97.3	6.50, d (2.1)	97.2	6.17, d (2.0)	100.0
7		166.3		166.3		165.9
8	6.62, d (2.1)	94.0	6.64, d (2.1)	94.0	6.31, d (2.0)	94.9
8a		161.8		161.8		159.9
9	2.63, t (7.5)	34.4	2.63, t (7.7)	34.3	2.64, t (7.6)	34.9
10	1.76, m	27.8	1.76, m	27.7	1.75, m	27.8
11	1.52, m	26.1	1.52, m	26.0	1.52, m	26.0
12	1.52, m	37.6	1.54, m	37.6	1.53, m	37.6
13	3.99, m	69.3	4.0, m	69.1	3.99, m	69.1
14	2.41, m	43.6	2.41, dd (15.1, 8.1) 2.50, dd (15.1, 4.9)	43.2	2.39, dd (15.3, 7.9) 2.46, dd (15.3, 5.1)	43.3
15		174.5		174.0		175.7
5-OCH_3_	3.89, s	56.5	3.89, s	56.5		
7-OCH_3_	3.90, s	52.0	3.91, s	56.5		
15-OCH_3_			3.67, s	52.0		

**Table 3:** ^1^H NMR Data of Compounds **7** and **8** in Methanol-*d*_4_ (δ in ppm, *J* in Hz).

Position	7		8	
	δ_H_	δ_C_	δ_H_	δ_C_
2		170.4		170.4
3	6.15, s	110.4	6.15, s	110.4
4		184.0		184.0
4a		106.2		106.2
5		163.0		163.0
6	6.32, d (1.9)	99.1	6.32, d (2.0)	99.1
7		167.3		167.3
8	6.52, d (1.9)	93.5	6.53, d (2.0)	93.4
8a		159.8		159.8
9	2.82, dd (14.3, 3.7) 2.65, dd (14.3, 8.4)	43.3	2.83, dd (14.3, 3.5) 2.66, dd (14.3, 8.8)	43.3
10	4.0, m	70.1	4.0, m	70.0
11	1.55, m	38.4	1.57, m	38.1
12	1.41, m	26.5	1.48, m 1.57, m	26.3
13	1.37, m	30.3	1.54, m	29.1
14	1.37, m	30.2	2.25, m	33.1
15	1.62, m	26.1	6.95, dd (15.7, 7.0)	150.6
16	2.28, t (7.4)	35.1	5.81, d (15.7)	123.0
17		177.9		170.5
5-OCH_3_				
7-OCH_3_	3.86, s	56.5	3.87, s	56.5

**Table 4:** The α-glucosidase inhibitory and antioxidant activities of compounds **1**–**10**.

Compounds	α-Glucosidase Inhibitory		Antioxidant	
	% Inhibition (667 μM)	IC_50_ (μM)	% Inhibition (1000 μM)	IC_50_ (μM)
**1**	−3.43		7.24	
**2**	15.10		35.7	1970 ± 156
**3**	23.78		20.4	
**4**	−9.718		12.2	
**5**	1.41		11.2	
**6**	27.3	842 ± 11	33.8	
**7**	25.2	917 ± 8	23.6	
**8**	41.7	1017 ± 22	19.3	
**9**	40.18	688 ± 24	18.1	
**10**	74.66	268 ± 25	21.8	
Acarbose ^a^	27.7	1330		
Vitamin C ^a^			96.4	26.7

^a^ Positive control.

## Data Availability

All data and figures in this study are openly available.

## Supplementary Materials

Figures S1–S55: ^1^H, ^13^C-NMR, HSQC, ^1^H-^1^H COSY, HMBC, HRESIMS spectra of new compounds **1**–**8**, and structures of reported analogs. Table S1: calculated specific rotations for **1**.

## Sample Availability

Samples of the compounds **1**–**10** are available from the authors.
