# Supplementary material for: Chromone Derivatives with α-Glucosidase Inhibitory Activity from the Marine Fungus Penicillium thomii Maire
Source: Molecules. 2021 Aug 31;26(17):5273. doi: 10.3390/molecules26175273 (PMC8434415; doi:10.3390/molecules26175273)
Supplement: Supplementary file 1 [file molecules-26-05273-s001.zip › molecules-1335355-supplementary.pdf]

Supplementary Material

Chromone Derivatives with α-Glucosidase Inhibitory Activity from the Marine Fungus *Penicillium thomii* Maire

Shouye Han [1], Yu Liu [1], Wan Liu [1], Fan Yang [1], Jia Zhang [1], Ruifeng Liu [1], Fenqin Zhao [1,*], Wei Xu [2,*] and Zhongbin Cheng [1,*]

[1] School of Pharmacy, Henan University, Kaifeng 475004, China; hanshouye123@163.com (S.H.); liuyu5230710@163.com (Y.L.); 18737806806@163.com (W.L.); Y18992588130@126.com (F.Y.); z919395@126.com (J.Z.); liurf12138@163.com (R.L.)

[2] Key Laboratory of Marine Biogenetic Resources, Third Institute of Oceanography, Ministry of Natural Resources, Xiamen 361005, China

[*] Correspondence: zhaofenqin2005@126.com (F.Z.); xuwei@tio.org.cn (W.X.); chengzhongbin@126.com (Z.C.); Tel.: +86-371-2388-3791 (Z.C.)

Table of Contents

		Page
**Figure S1**	^{1}H NMR Spectrum of **1** in DMSO-*d*$_6$ (400 MHz).	1
**Figure S2**	^{13}C NMR Spectrum of **1** in DMSO-*d*$_6$ (100 MHz)	1
**Figure S3**	HSQC Spectrum of **1** in DMSO-*d*$_6$	2
**Figure S4**	^{1}H-^{1}H COSY Spectrum of **1** in DMSO-*d*$_6$	2
**Figure S5**	HMBC Spectrum of **1** in DMSO-*d*$_6$	3
**Figure S6**	NOESY Spectrum of **1** in DMSO-*d*$_6$	3
**Figure S7**	^{1}H NMR Spectrum of **2** in Methanol-*d*$_4$ (400 MHz)	4
**Figure S8**	^{13}C NMR Spectrum of **2** in Methanol-*d*$_4$ (100 MHz)	4
**Figure S9**	HSQC Spectrum of **2** in Methanol-*d*$_4$	5
**Figure S10**	^{1}H-^{1}H COSY Spectrum of **2** in Methanol-*d*$_4$	5
**Figure S11**	HMBC Spectrum of **2** in Methanol-*d*$_4$	6
**Figure S12**	NOESY Spectrum of **2** in Methanol-*d*$_4$	6
**Figure S13**	^{1}H NMR Spectrum of **3** in Methanol-*d*$_4$ (400 MHz).	7
**Figure S14**	^{13}C NMR Spectrum of **3** in Methanol-*d*$_4$ (100 MHz)	7
**Figure S15**	HSQC Spectrum of **3** in Methanol-*d*$_4$	8
**Figure S16**	^{1}H-^{1}H COSY Spectrum of **3** in Methanol-*d*$_4$	8
**Figure S17**	HMBC Spectrum of **3** in Methanol-*d*$_4$	9
**Figure S18**	NOESY Spectrum of **3** in Methanol-*d*$_4$	9
**Figure S19**	^{1}H NMR Spectrum of **4** in Methanol-*d*$_4$ (400 MHz).	10
**Figure S20**	^{13}C NMR Spectrum of **4** in Methanol-*d*$_4$ (100 MHz)	10
**Figure S21**	HSQC Spectrum of **4** in Methanol-*d*$_4$	11
**Figure S22**	^{1}H-^{1}H COSY Spectrum of **4** in Methanol-*d*$_4$	11
**Figure S23**	HMBC Spectrum of **4** in Methanol-*d*$_4$	12
**Figure S24**	NOESY Spectrum of **4** in Methanol-*d*$_4$	12
**Figure S25**	^{1}H NMR Spectrum of **5** in Methanol-*d*$_4$ (400 MHz).	13
**Figure S26**	^{13}C NMR Spectrum of **5** in Methanol-*d*$_4$ (100 MHz)	13
**Figure S27**	HSQC Spectrum of **5** in Methanol-*d*$_4$	14
**Figure S28**	^{1}H-^{1}H COSY Spectrum of **5** in Methanol-*d*$_4$	14
**Figure S29**	HMBC Spectrum of **5** in Methanol-*d*$_4$	15
**Figure S30**	NOESY Spectrum of **5** in Methanol-*d*$_4$	15
**Figure S31**	^{1}H NMR Spectrum of **6** in Methanol-*d*$_4$ (400 MHz).	16
**Figure S32**	^{13}C NMR Spectrum of **6** in Methanol-*d*$_4$ (100 MHz)	16
**Figure S33**	HSQC Spectrum of **6** in Methanol-*d*$_4$	17
**Figure S34**	^{1}H-^{1}H COSY Spectrum of **6** in Methanol-*d*$_4$	17
**Figure S35**	HMBC Spectrum of **6** in Methanol-*d*$_4$	18
**Figure S36**	NOESY Spectrum of **6** in Methanol-*d*$_4$	18
**Figure S37**	^{1}H NMR Spectrum of **7** in Methanol-*d*$_4$ (400 MHz)	19
**Figure S38**	^{13}C NMR Spectrum of **7** in Methanol-*d*$_4$ (100 MHz)	19
**Figure S39**	HSQC Spectrum of **7** in Methanol-*d*$_4$	20
**Figure S40**	^{1}H-^{1}H COSY Spectrum of **7** in Methanol-*d*$_4$	20
**Figure S41**	HMBC Spectrum of **7** in Methanol-*d*$_4$	21
**Figure S42**	^{1}H NMR Spectrum of **8** in Methanol-*d*$_4$ (400 MHz)	21
**Figure S43**	^{13}C NMR Spectrum of **8** in Methanol-*d*$_4$ (100 MHz)	22
**Figure S44**	HSQC Spectrum of **8** in Methanol-*d*$_4$	22
**Figure S45**	^{1}H-^{1}H COSY Spectrum of **8** in Methanol-*d*$_4$	23
**Figure S46**	HMBC Spectrum of **8** in Methanol-*d*$_4$	23

**Figure S47**	HRESIMS Spectrum of **1**	24
**Figure S48**	HRESIMS Spectrum of **2**	25
**Figure S49**	HRESIMS Spectrum of **3**	26
**Figure S50**	HRESIMS Spectrum of **4**	27
**Figure S51**	HRESIMS Spectrum of **5**	28
**Figure S52**	HRESIMS Spectrum of **6**	29
**Figure S53**	HRESIMS Spectrum of **7**	30
**Figure S54**	HRESIMS Spectrum of **8**	30
**Figure S55**	Structures of Reported Analogs	31
**Table S1**	Calculation of the Specific Rotations	31

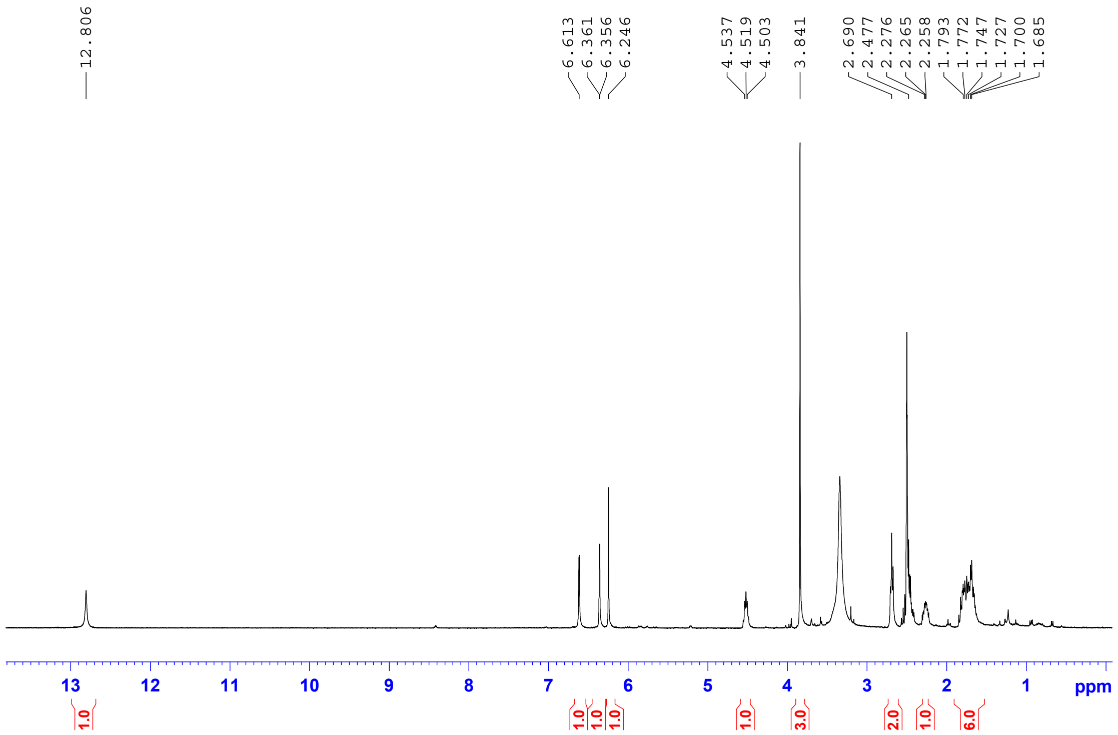

Figure S1. ^{1}H NMR Spectrum of **1** in Methanol-d_4 (400 MHz).

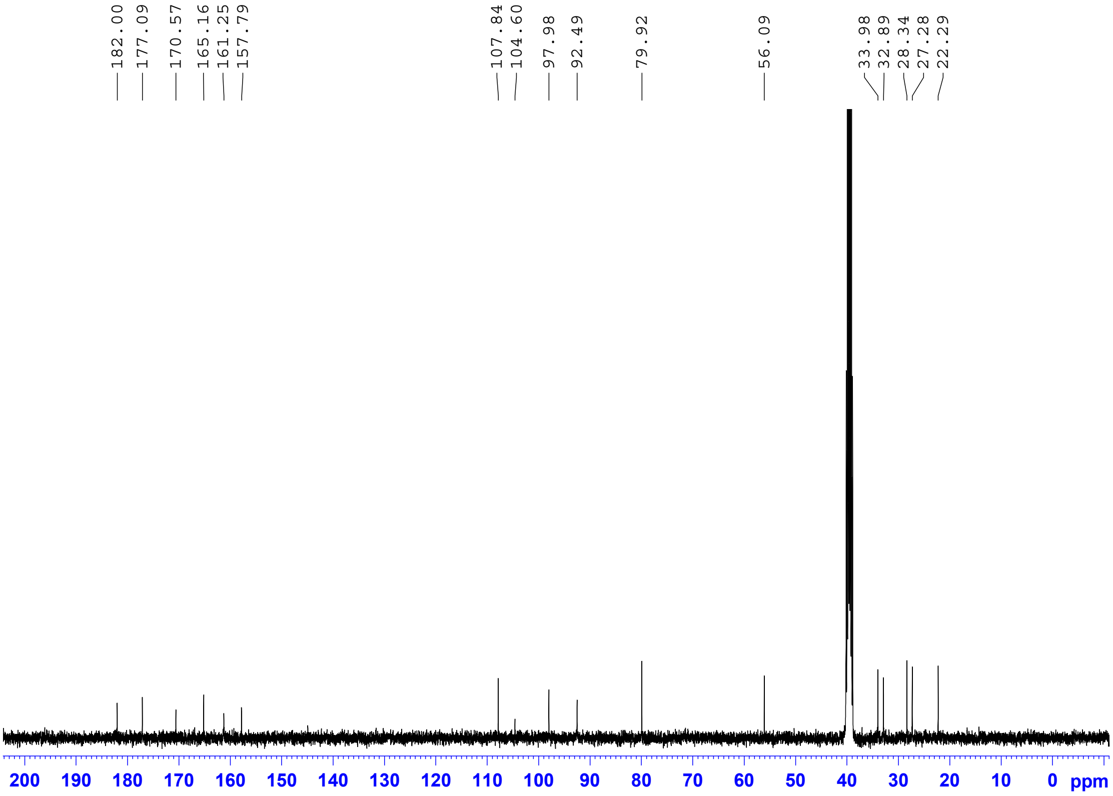

Figure S2. ^{13}C NMR Spectrum of **1** in Methanol-d_4 (100 MHz).

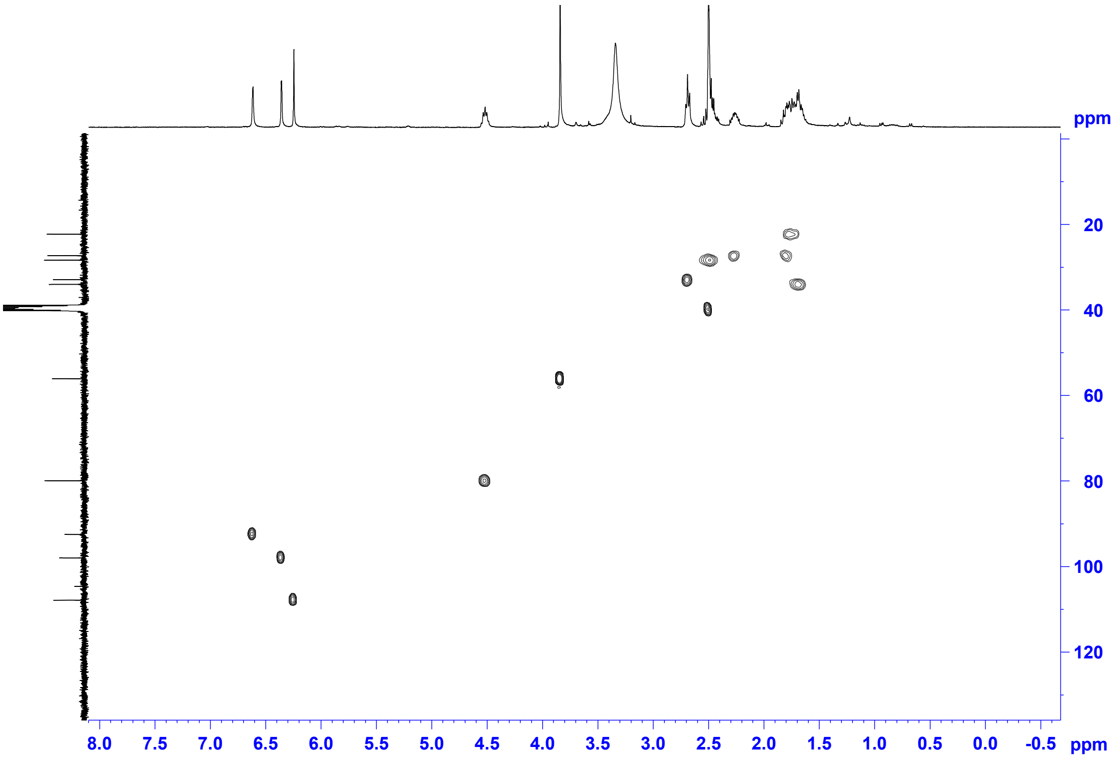

Figure S3. HSQC Spectrum of **1** in Methanol-d4

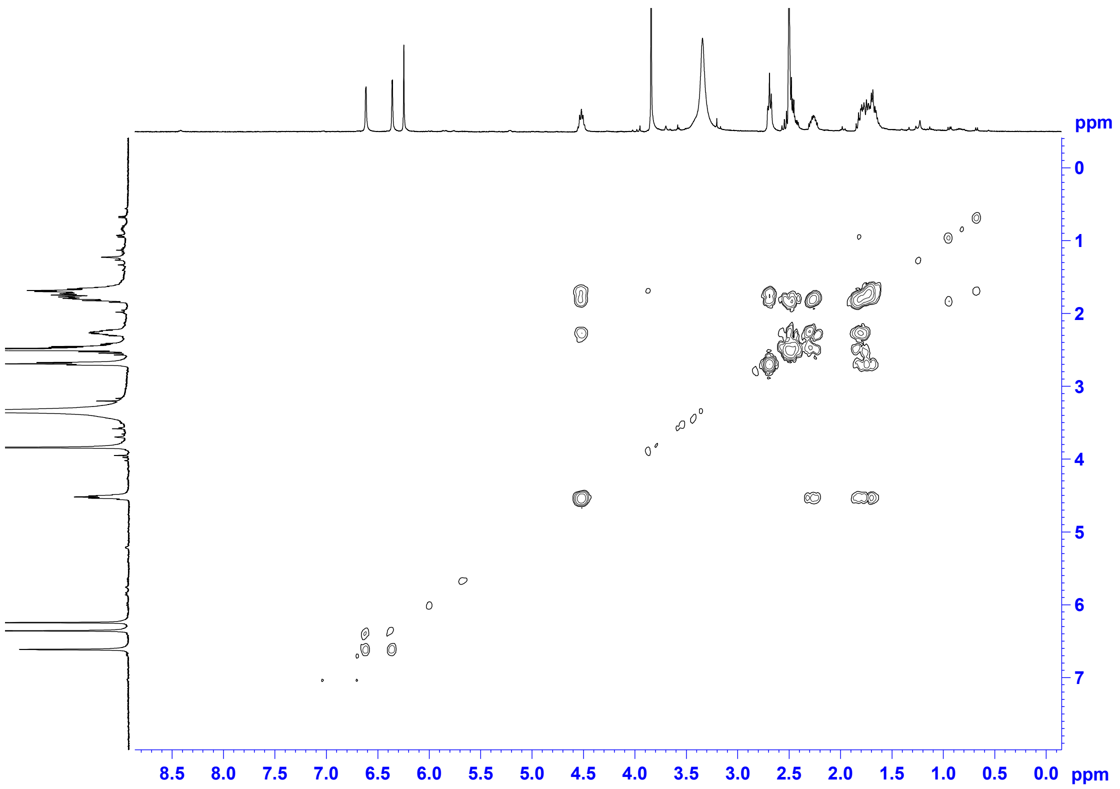

Figure S4. ^{1}H-^{1}H COSY Spectrum of **1** in Methanol-d4.

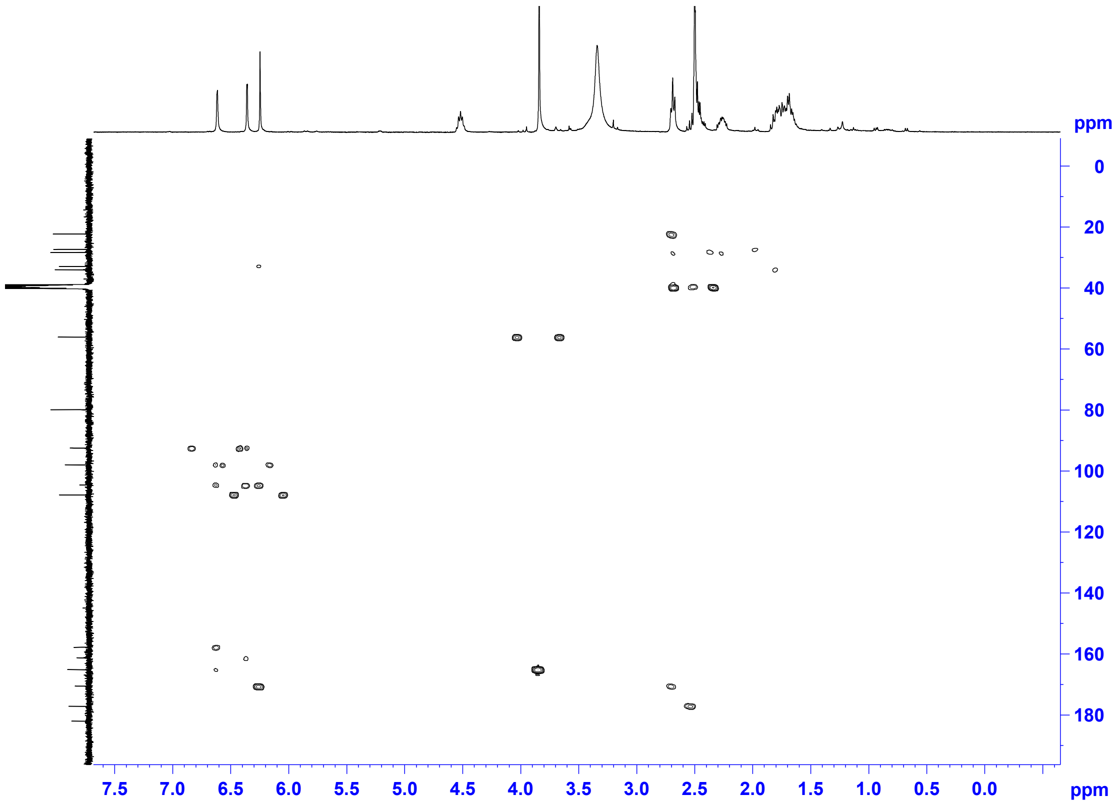

Figure S5. HMBC Spectrum of **1** in Methanol-d4.

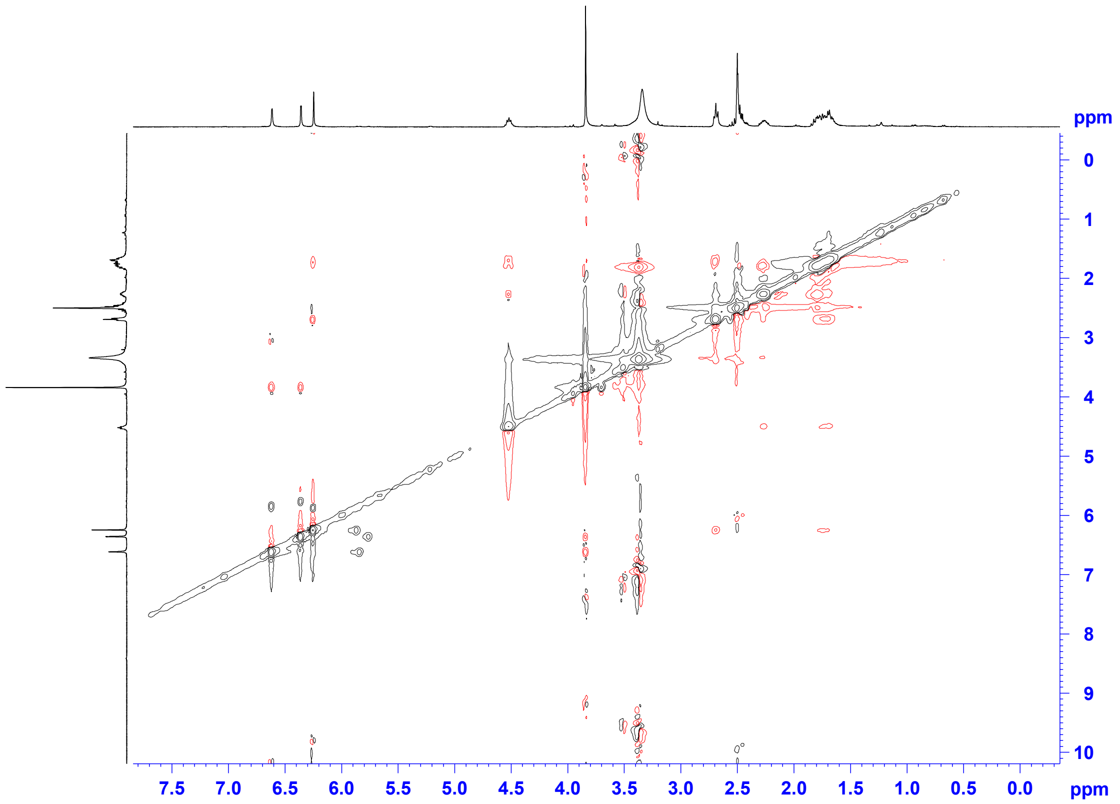

Figure S6. NOESY Spectrum of **1** in Methanol-d4.

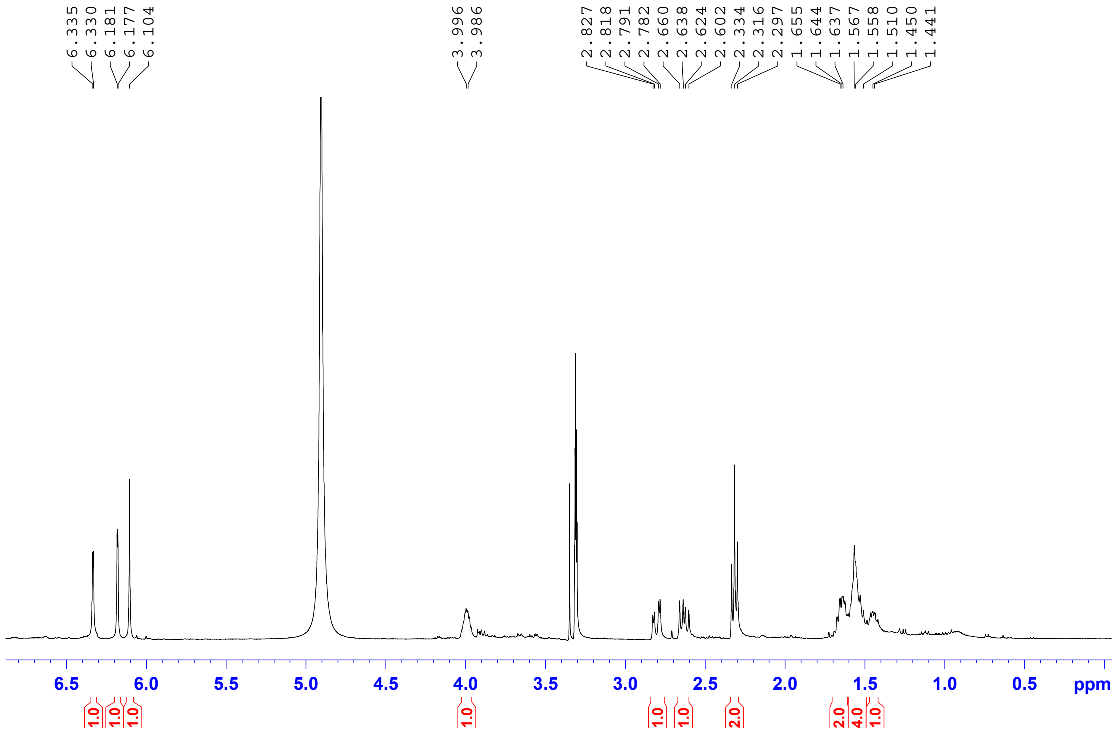

Figure S7. ^{1}H NMR Spectrum of **2** in Methanol-d_4 (400 MHz).

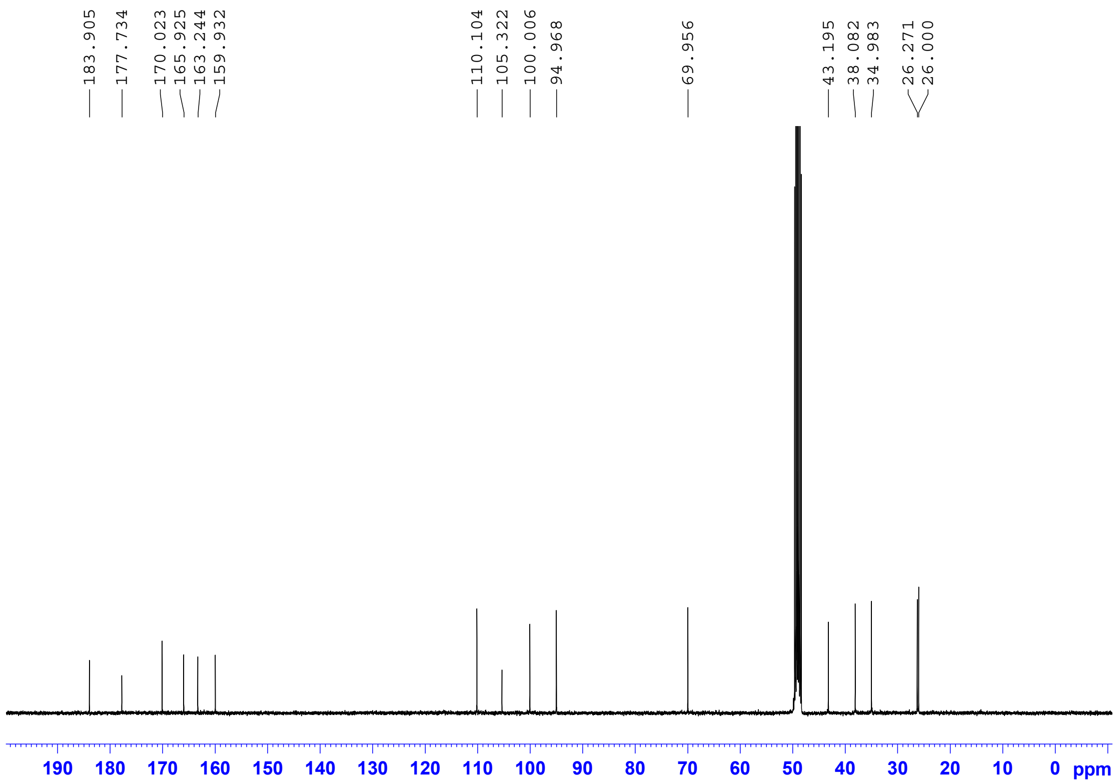

Figure S8. ^{13}C NMR Spectrum of **2** in Methanol-d_4 (100 MHz).

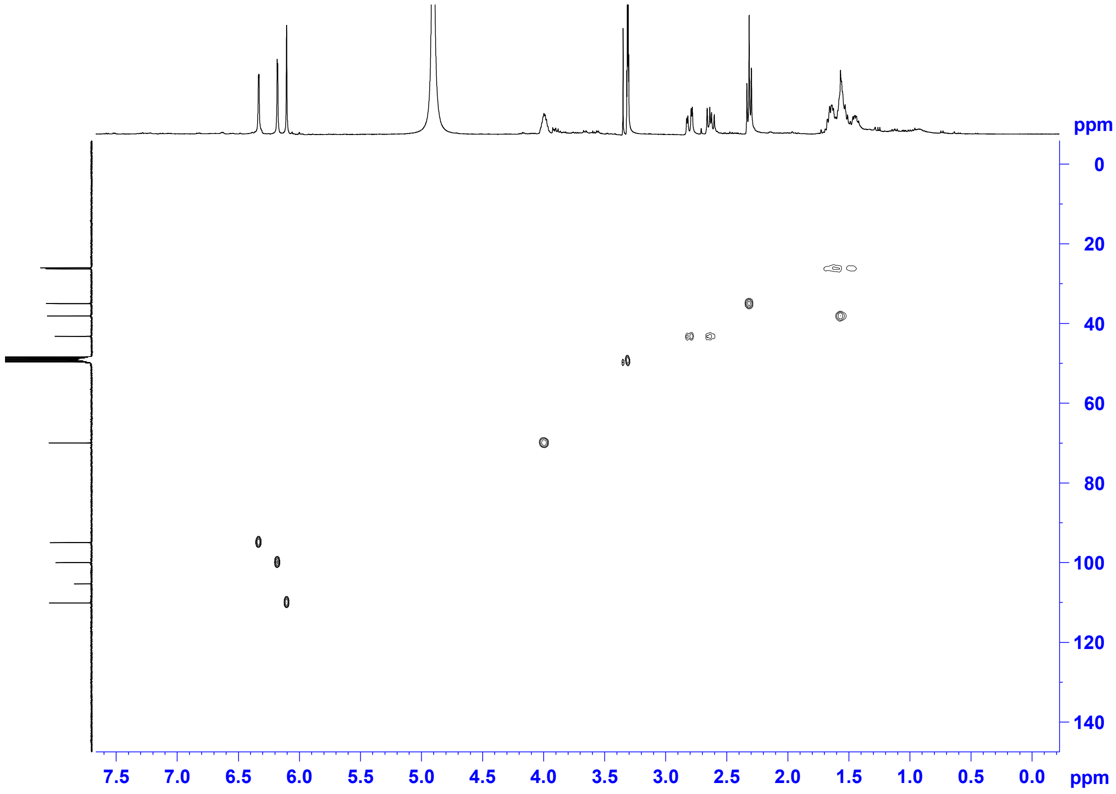

Figure S9. HSQC Spectrum of **2** in Methanol-d_4

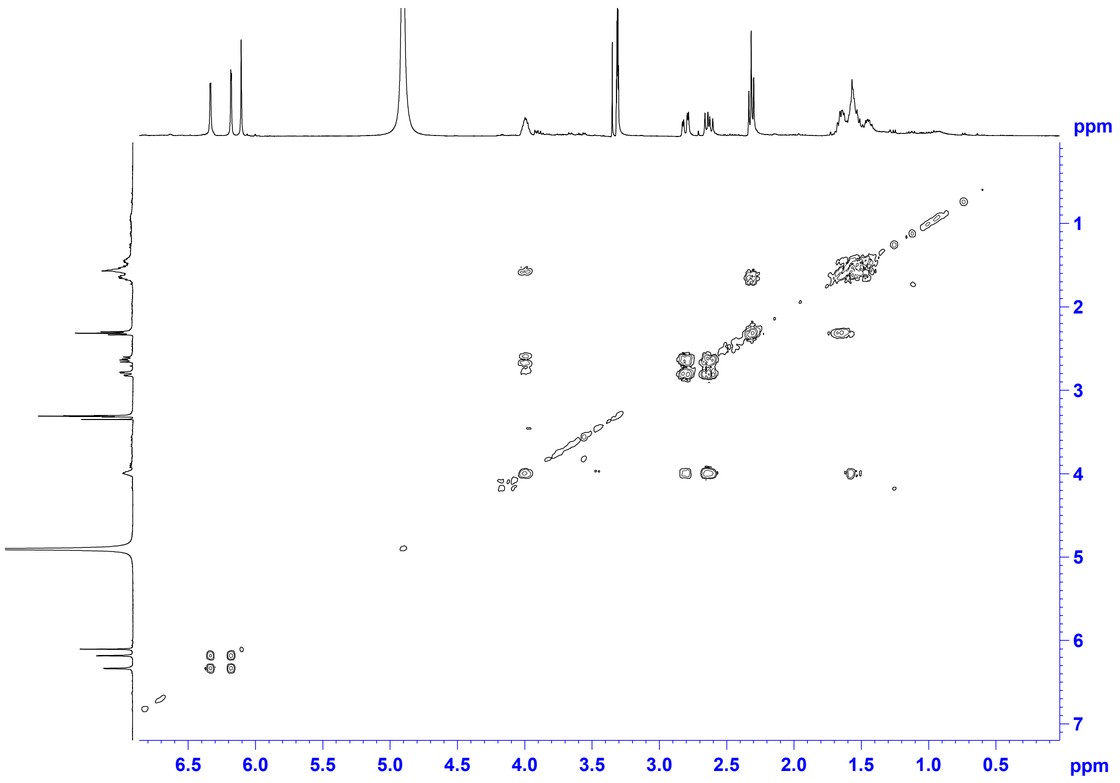

Figure S10. ^{1}H-^{1}H COSY Spectrum of **2** in Methanol-*d*4.

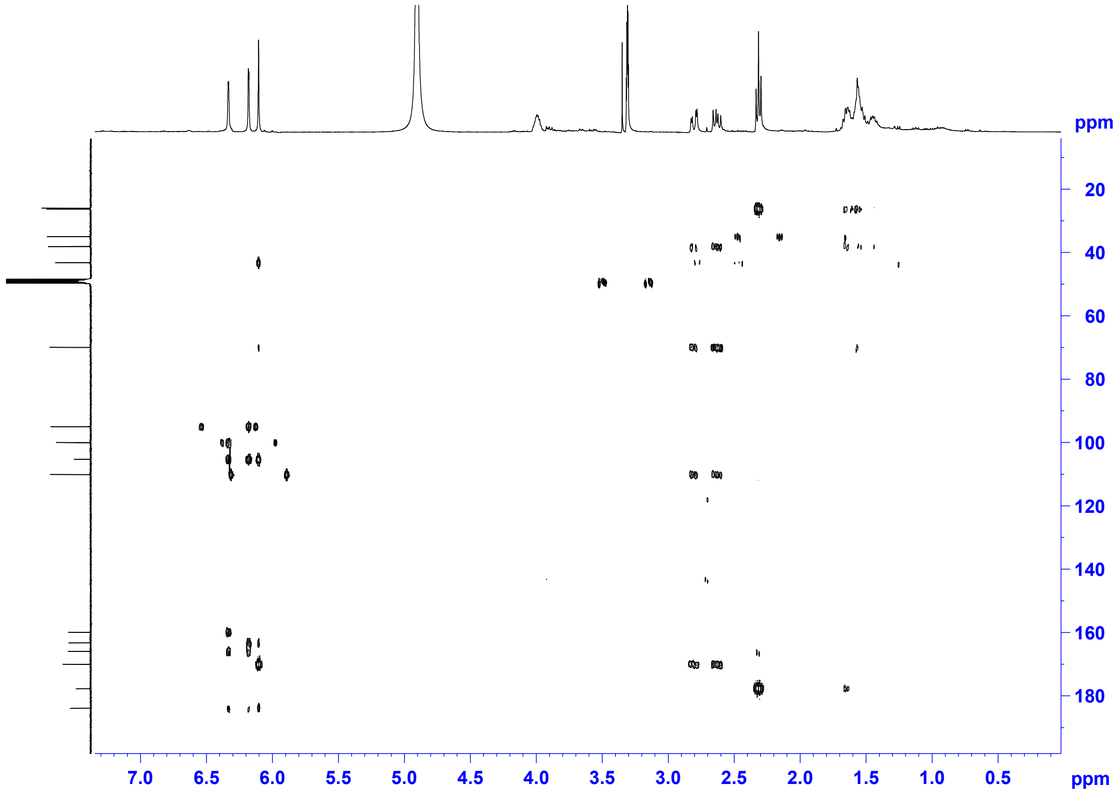

Figure S11. HMBC Spectrum of **2** in Methanol-*d*4

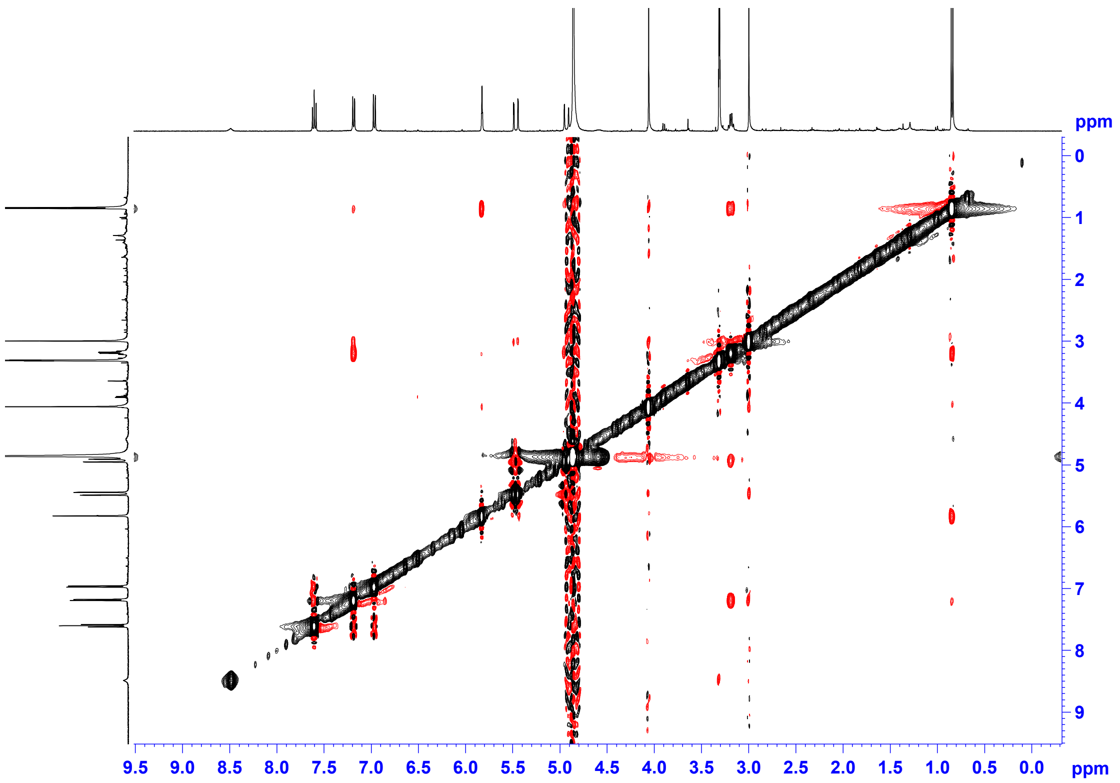

Figure S12. NOESY Spectrum of **2** in Methanol-d_4.

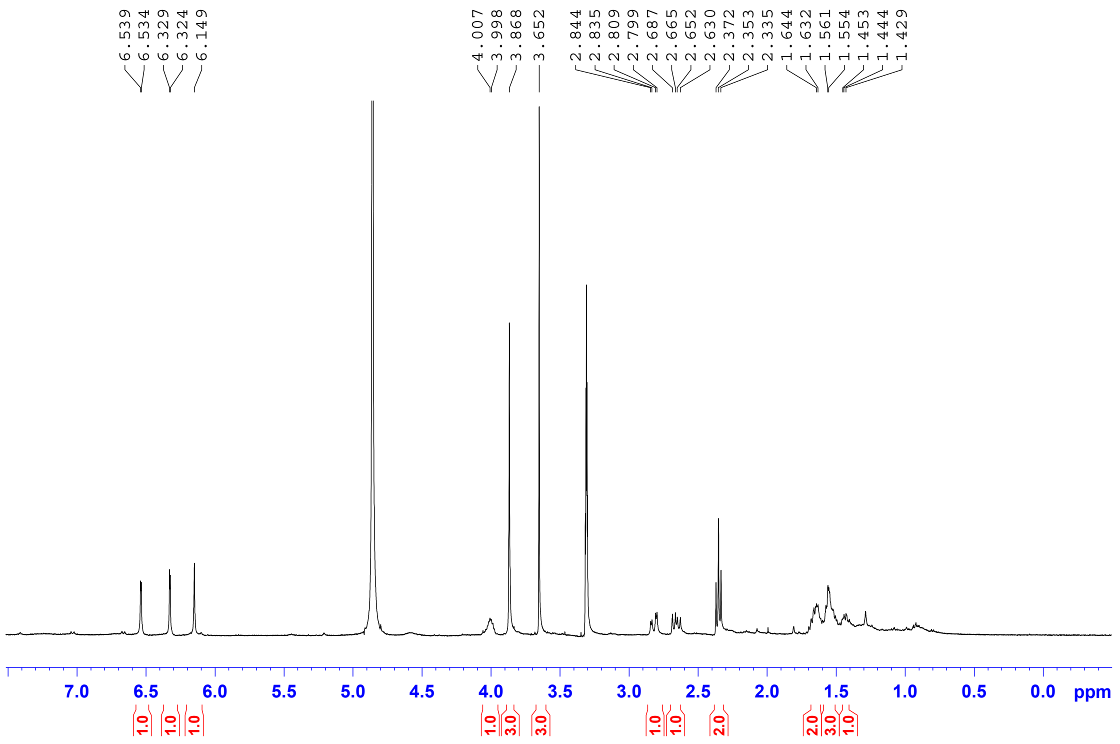

Figure S13. ^{1}H NMR Spectrum of **3** in Methanol-d_4 (400 MHz)

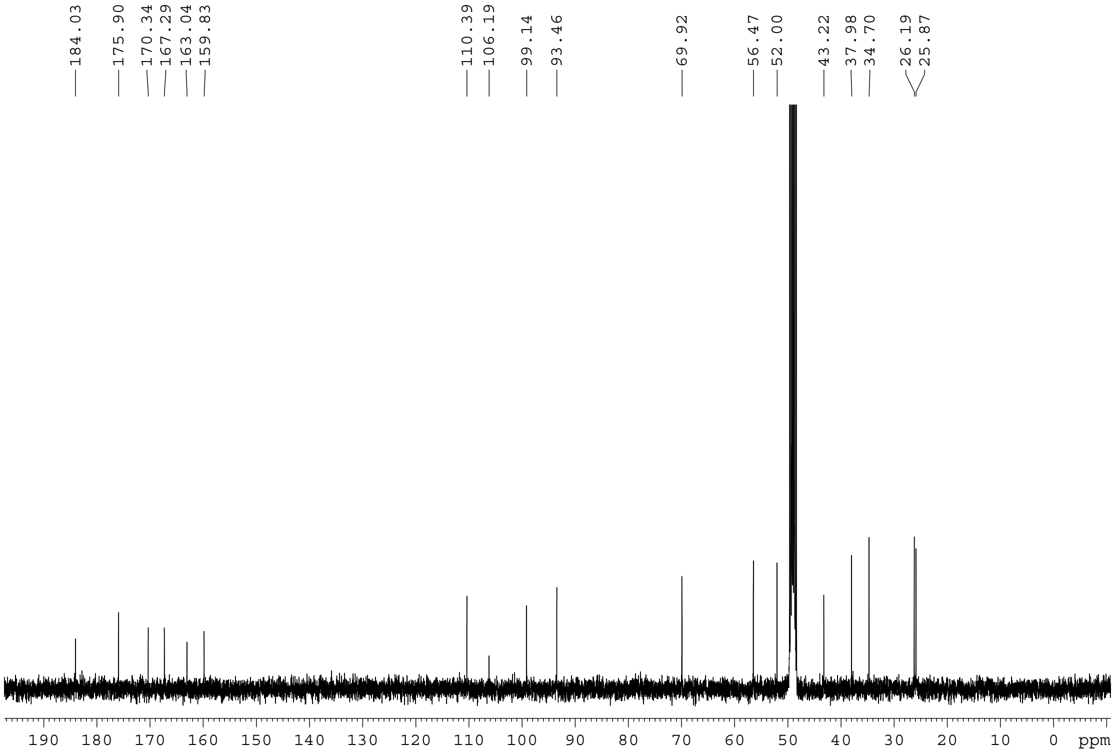

Figure S14. ^{13}C NMR Spectrum of **3** in Methanol-d_4 (100 MHz).

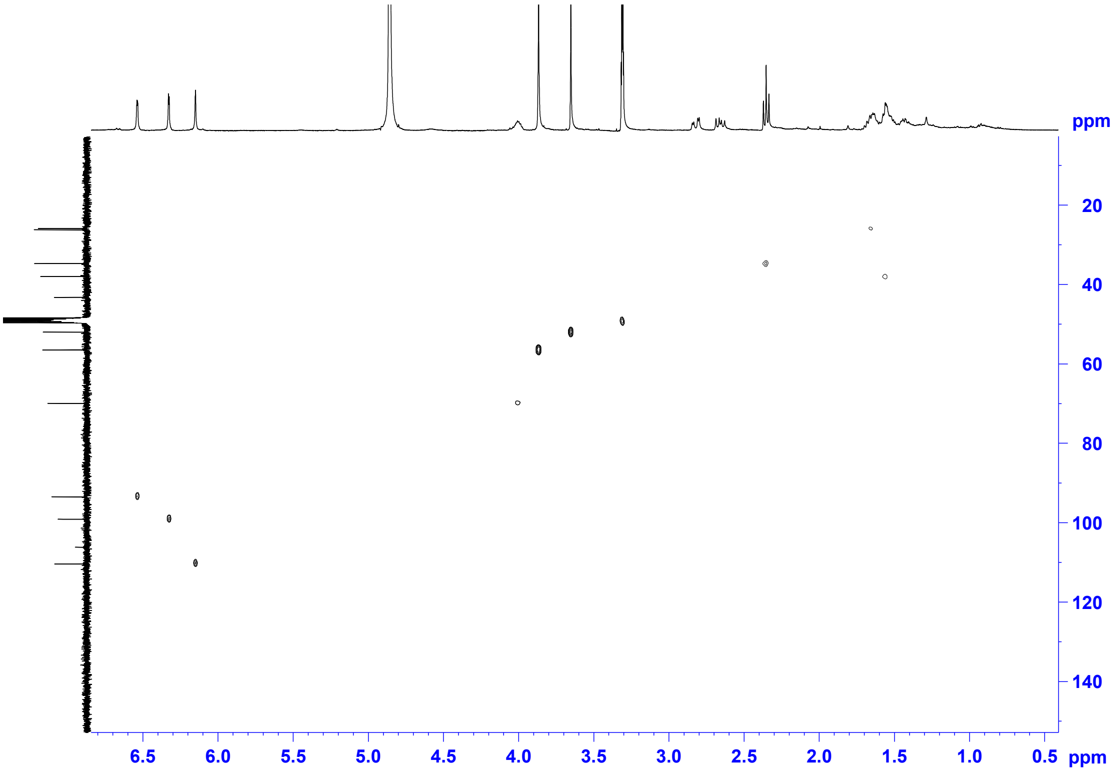

Figure S15. HSQC Spectrum of **3** in Methanol-d_4.

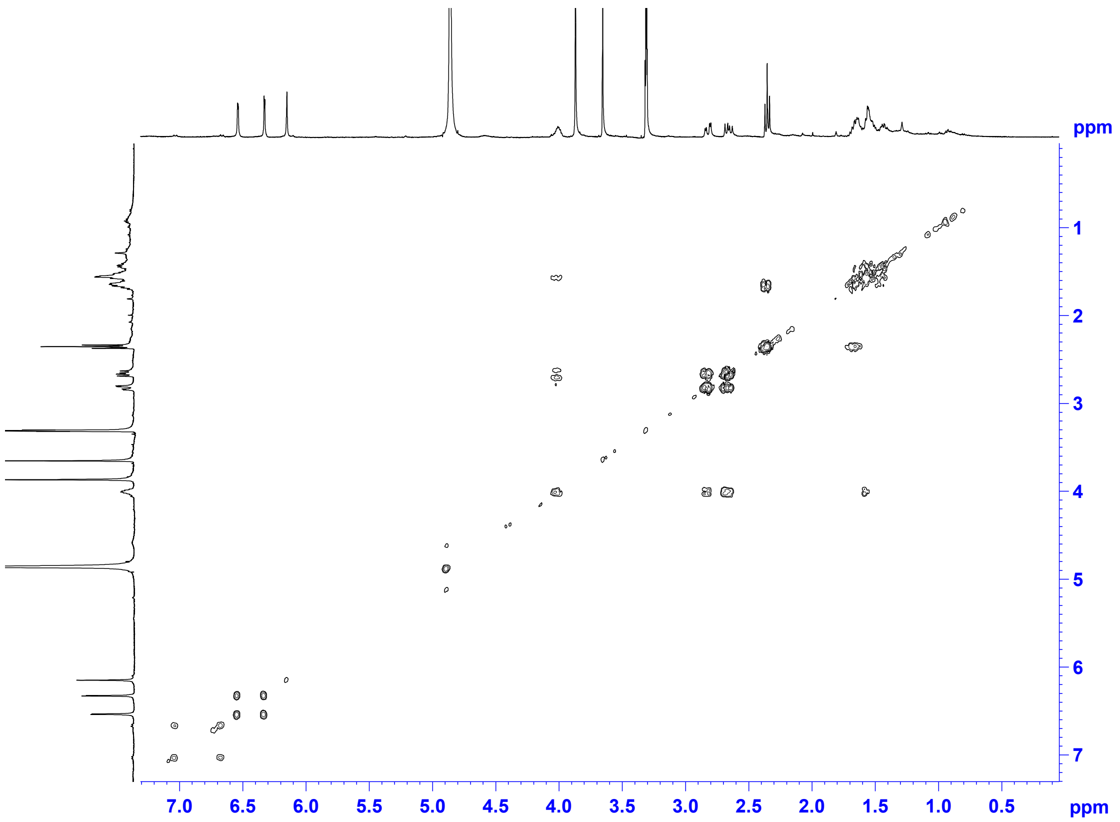

Figure S16. ^{1}H-^{1}H COSY Spectrum of **3** in Methanol-*d*4.

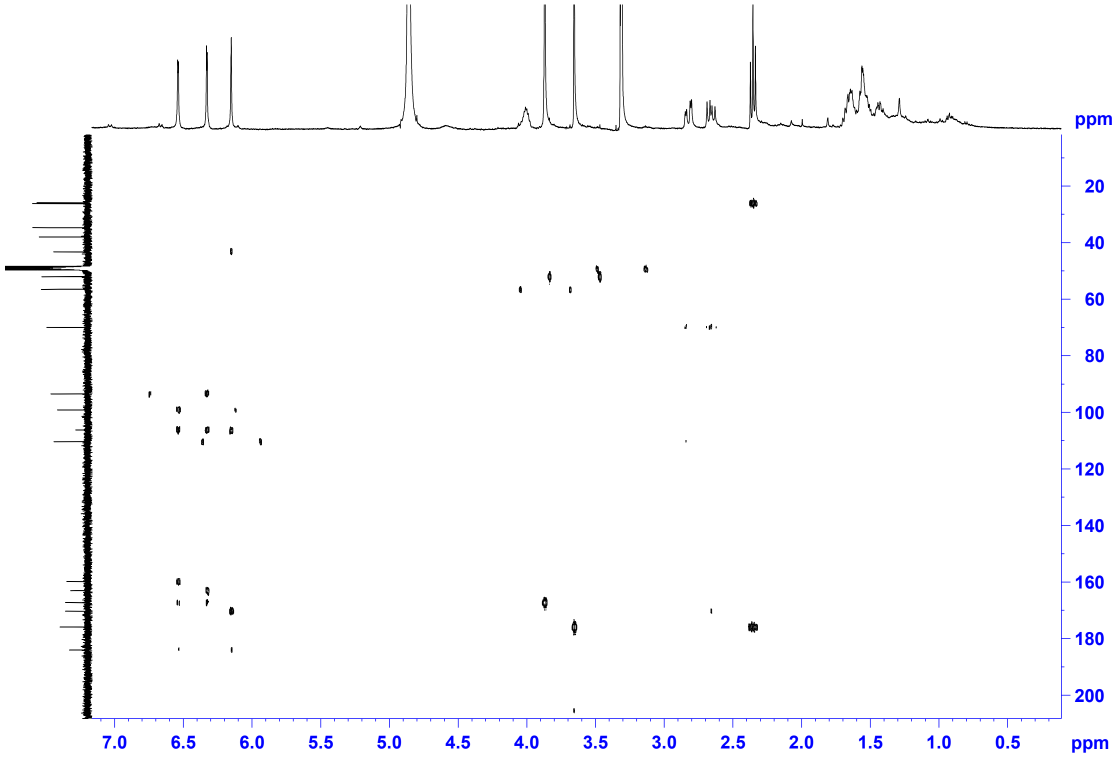

Figure S17. HMBC Spectrum of **3** in Methanol-*d*4.

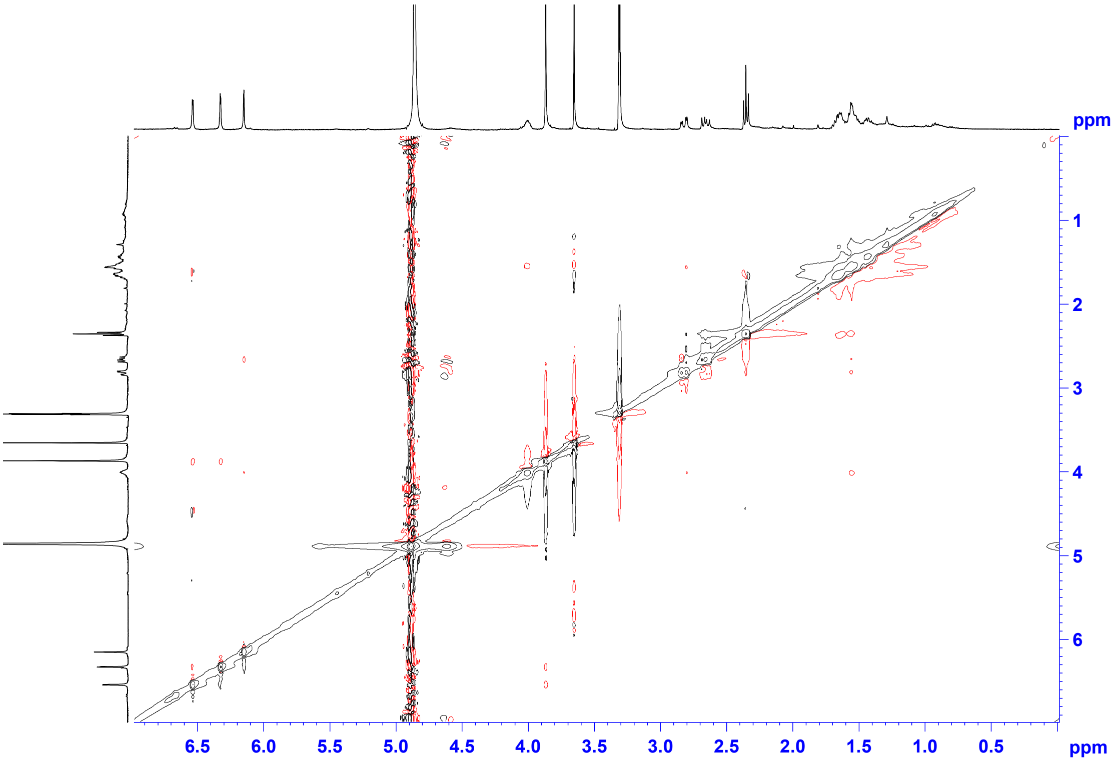

Figure S18. NOESY Spectrum of **3** in Methanol-d_4.

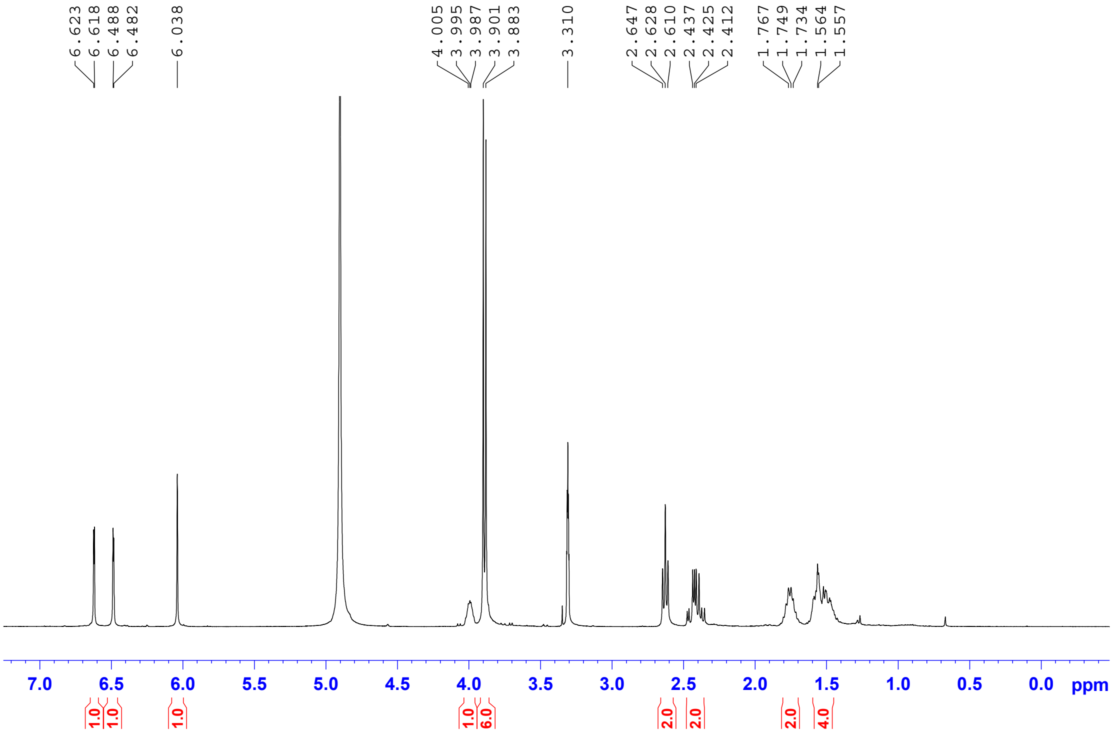

Figure S19. ^{1}H NMR Spectrum of **4** in Methanol-d_4 (400 MHz).

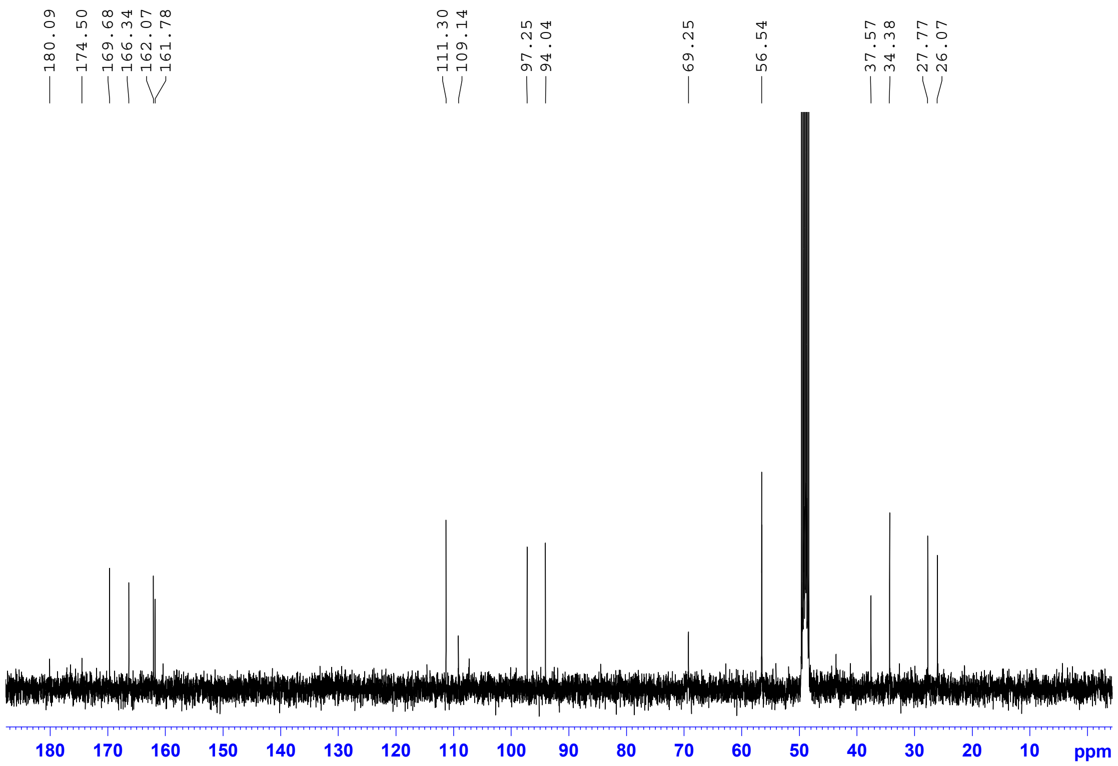

Figure S20. ^{13}C NMR Spectrum of **4** in Methanol-d_4 (100 MHz).

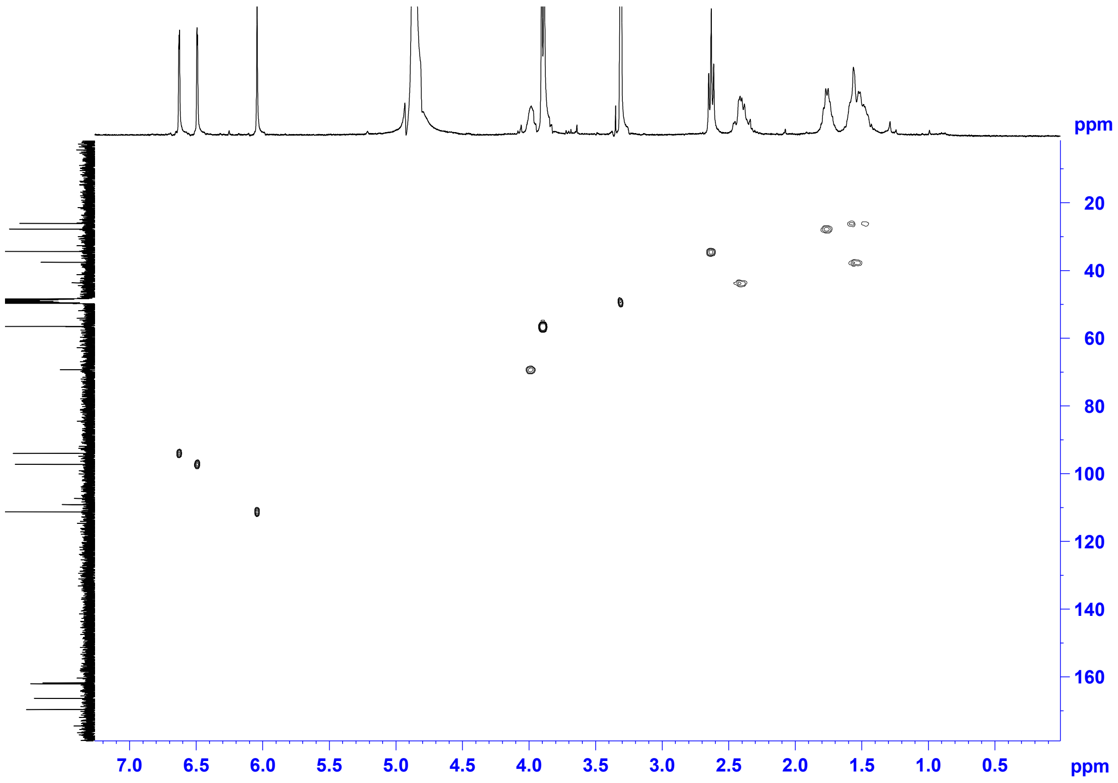

Figure S21. HSQC Spectrum of **4** in Methanol-d_4.

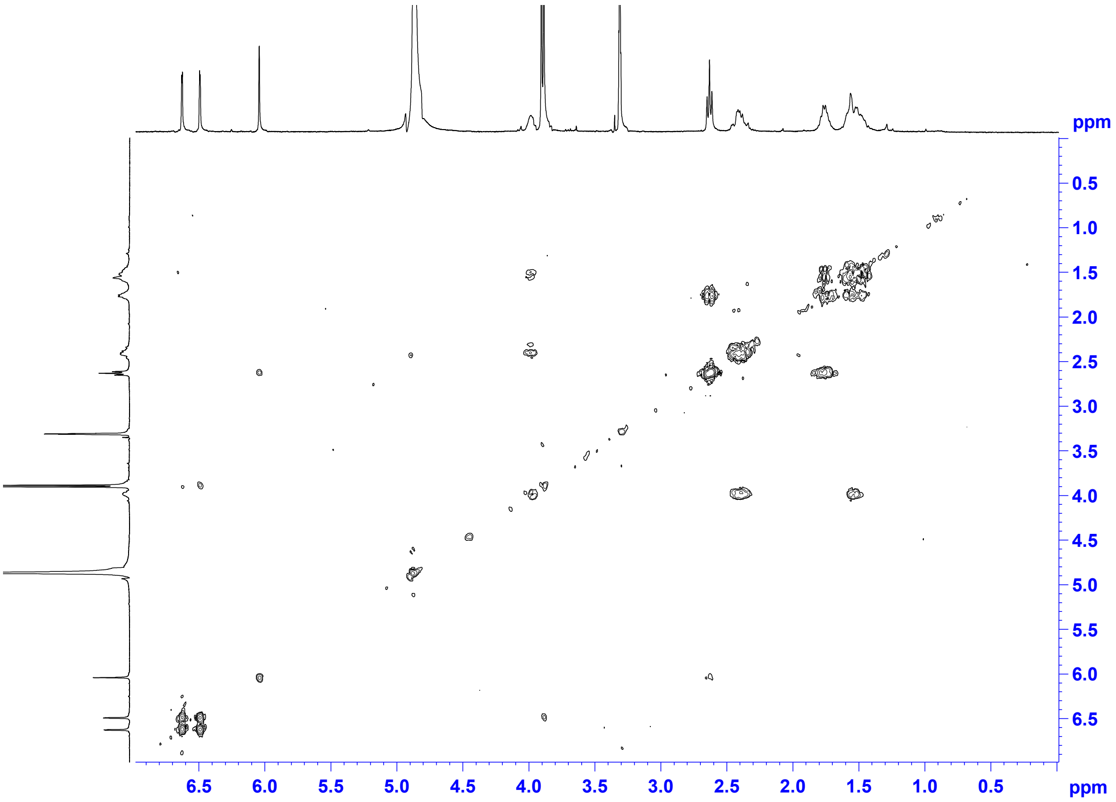

Figure S22. ^{1}H-^{1}H COSY Spectrum of **4** in Methanol-d_4.

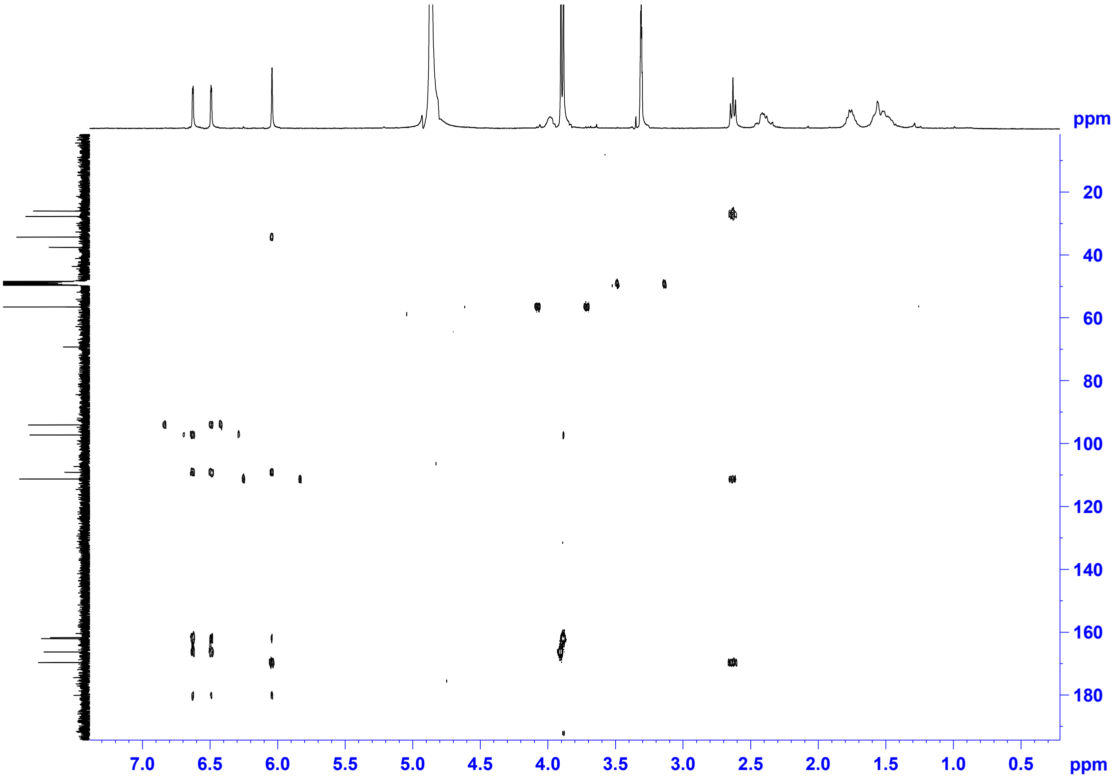

Figure S23. HMBC Spectrum of **4** in Methanol-d_4.

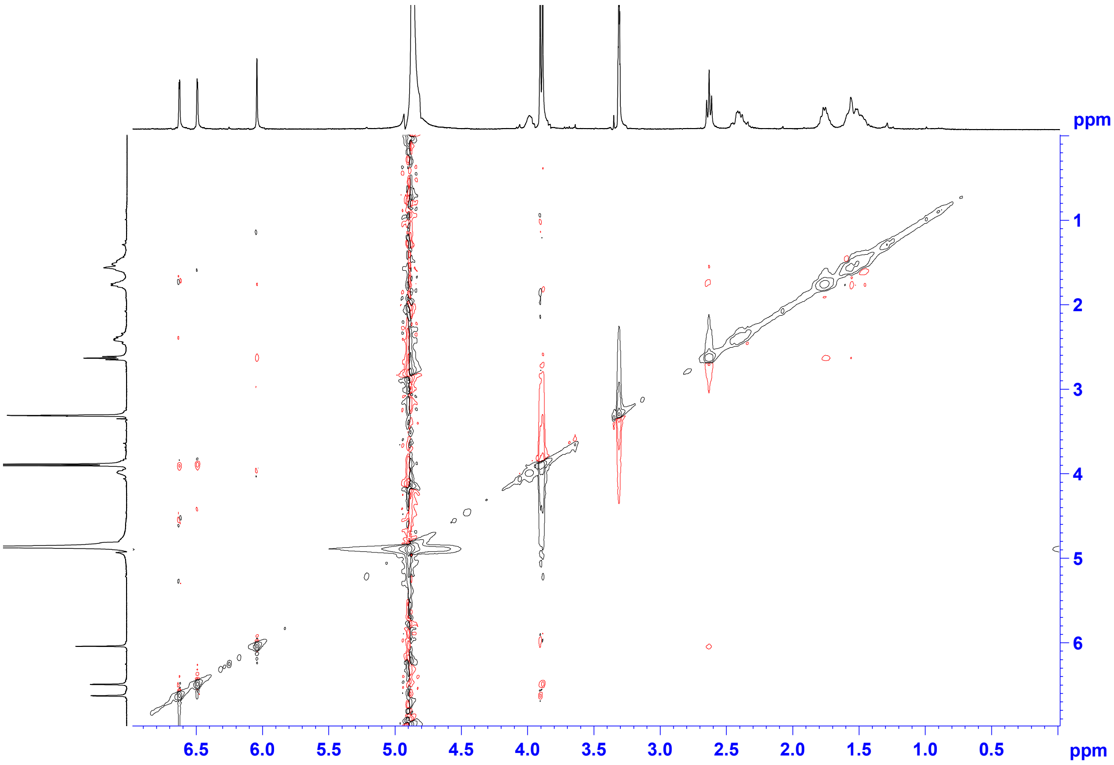

Figure S24. NOESY Spectrum of **4** in Methanol-d_4.

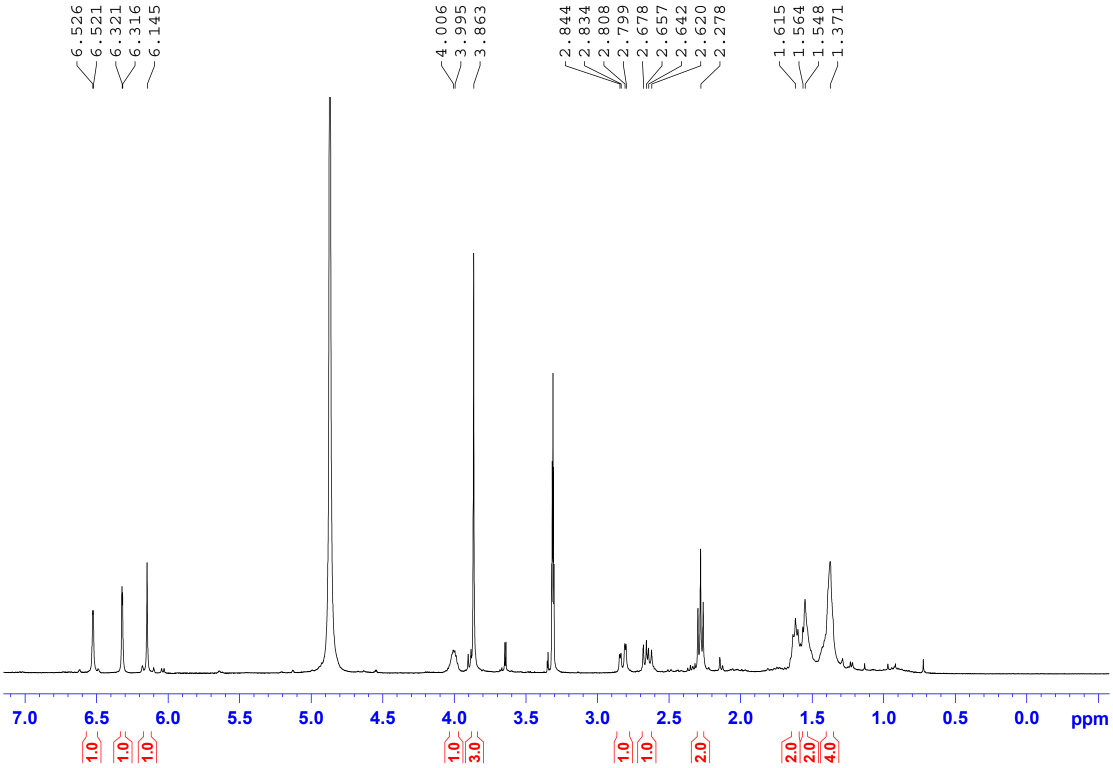

Figure S25. ^{1}H NMR Spectrum of **5** in Methanol-d_4 (400 MHz).

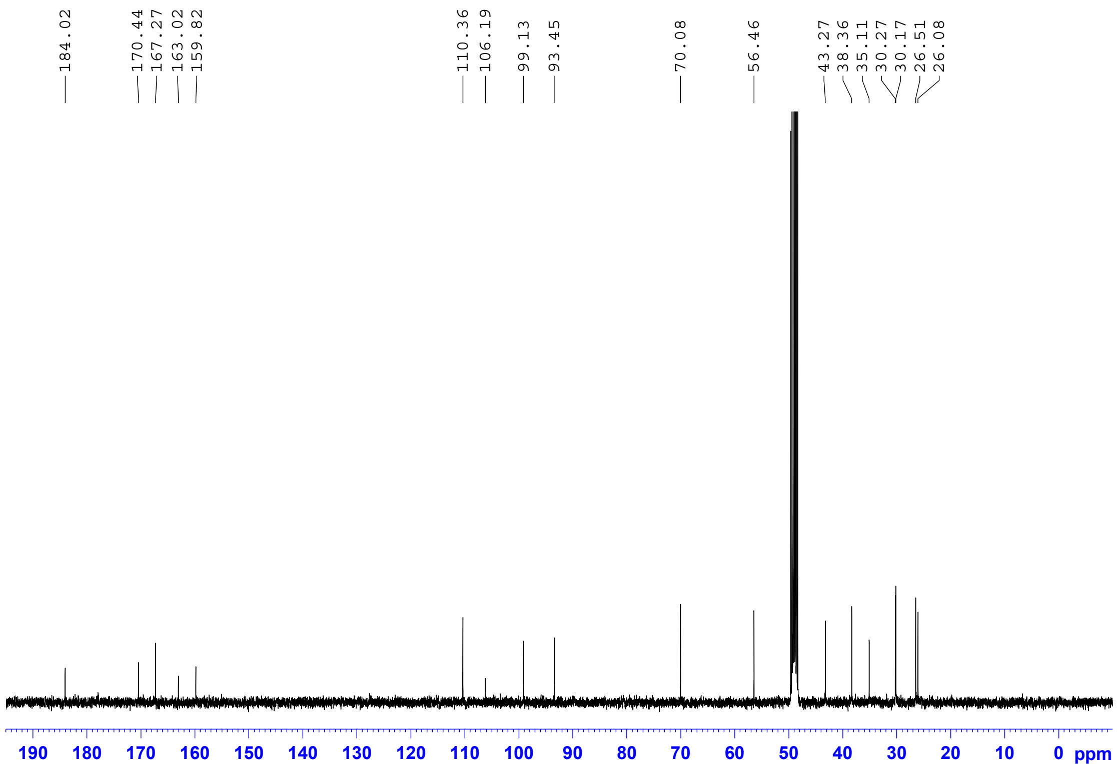

Figure S26. ^{13}C NMR Spectrum of **5** in Methanol-d_4 (100 MHz).

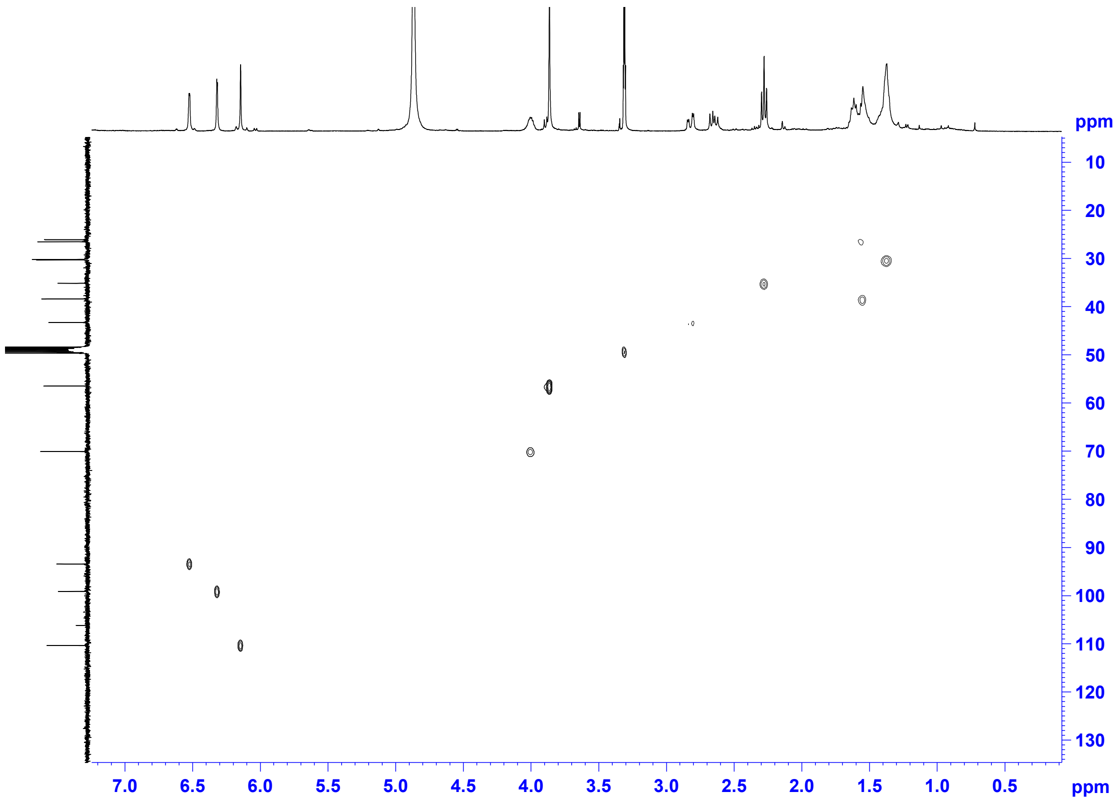

Figure S27. HSQC Spectrum of **5** in Methanol-d_4.

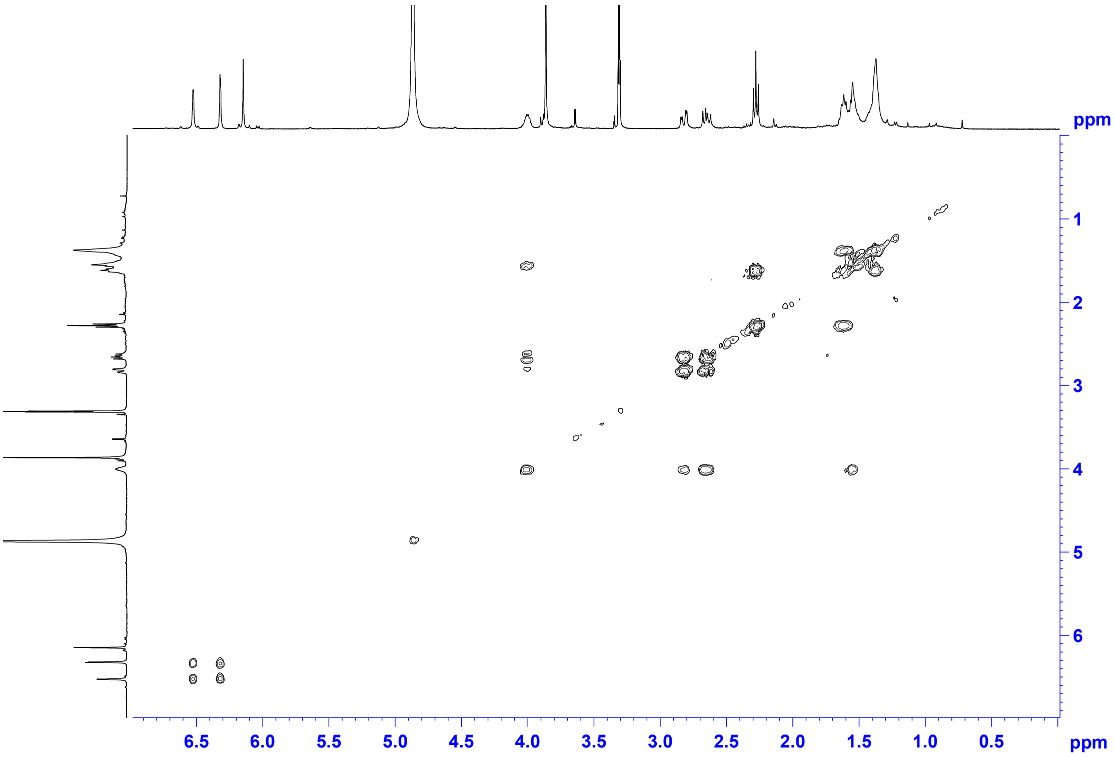

Figure S28. ^1H-^1H COSY Spectrum of **5** in Methanol-d_4.

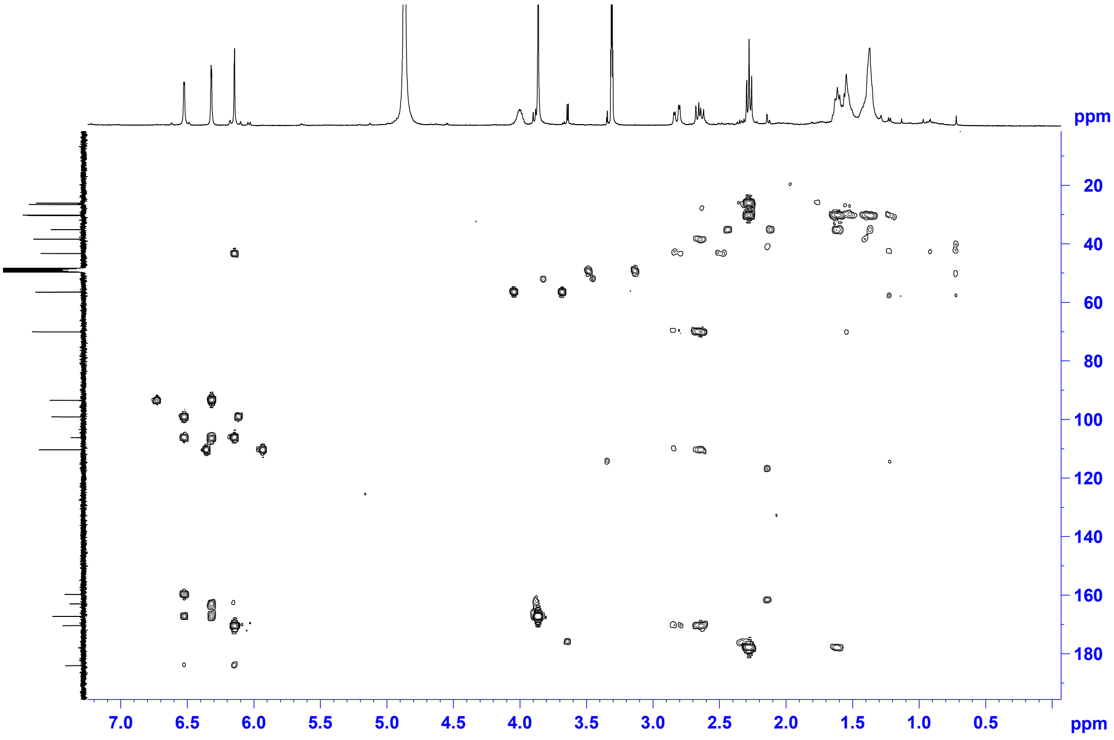

Figure S29. HMBC Spectrum of **5** in Methanol-d_4.

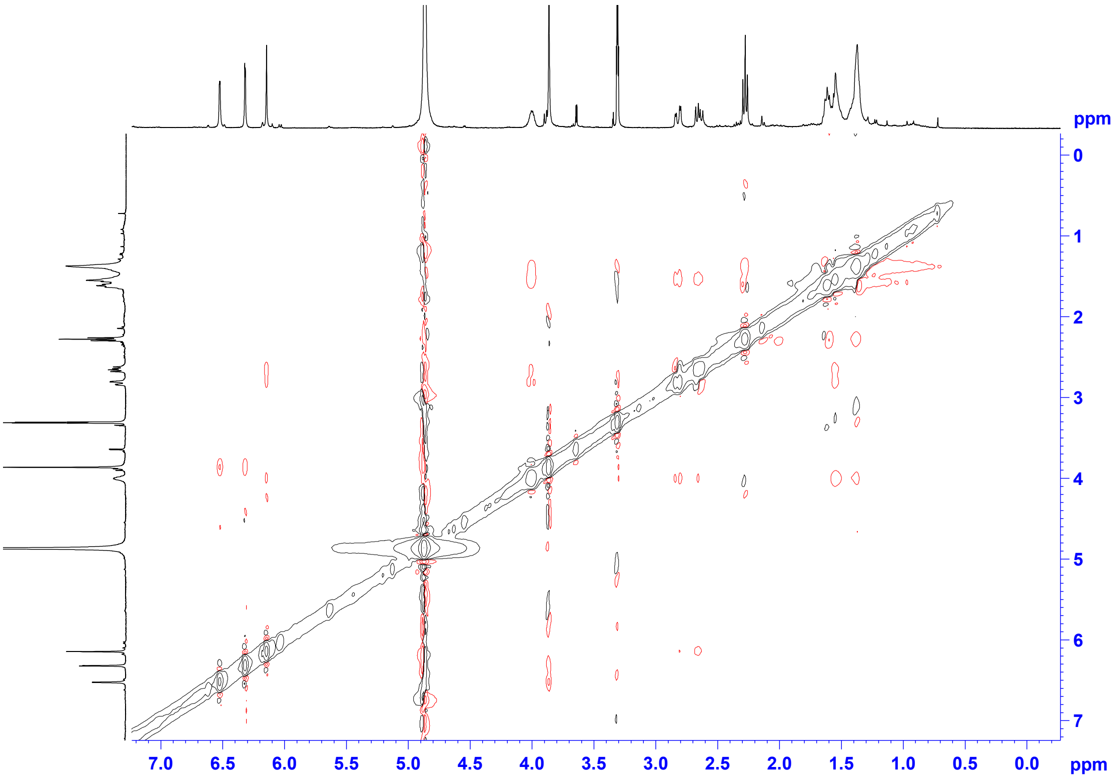

Figure S30. NOESY Spectrum of **5** in Methanol-d_4.

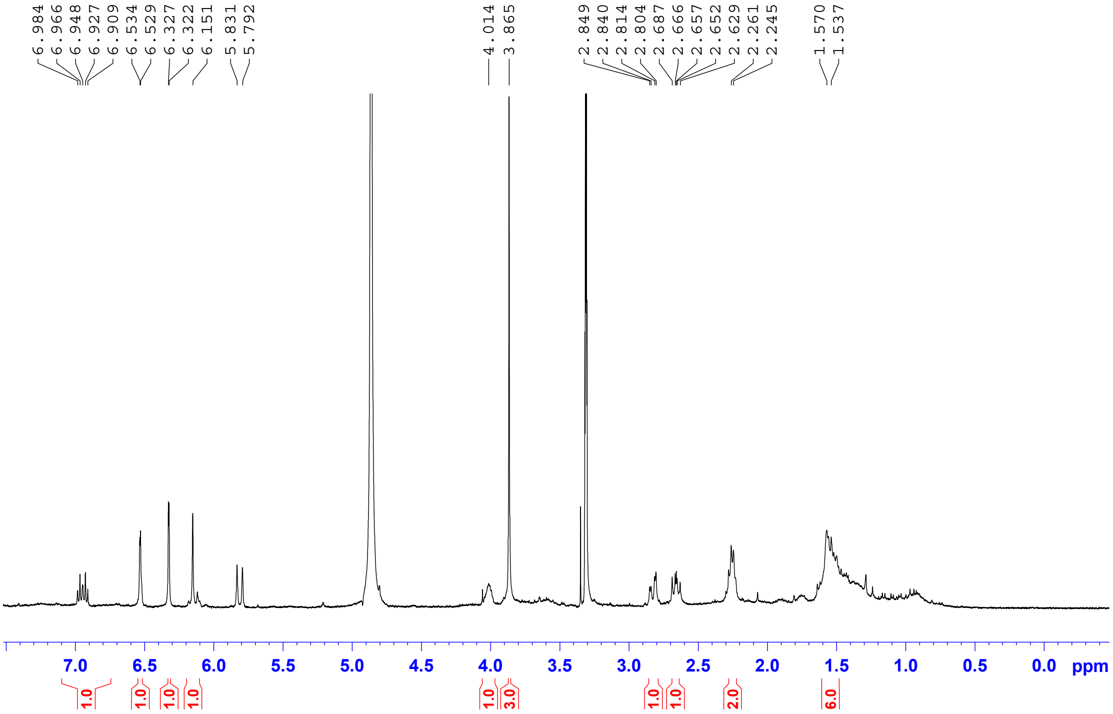

Figure S31. ^{1}H NMR Spectrum of **6** in Methanol-d_4 (400 MHz).

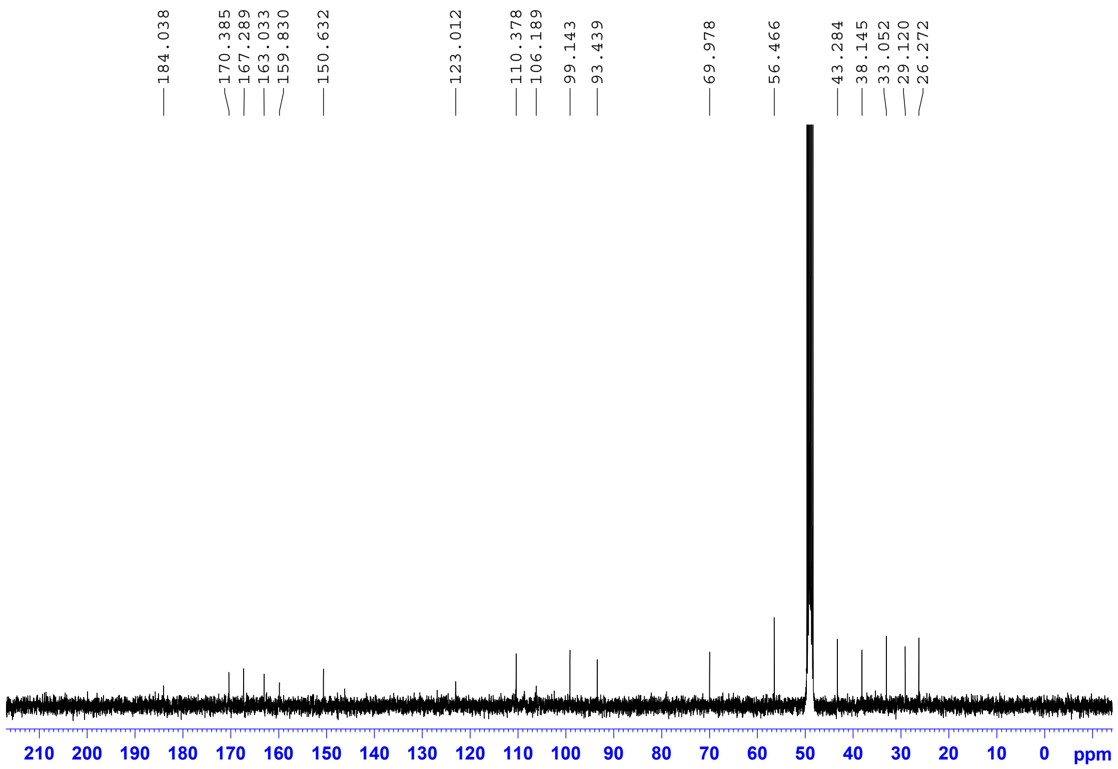

Figure S32. ^{13}C NMR Spectrum of **6** in Methanol-d_4 (100 MHz).

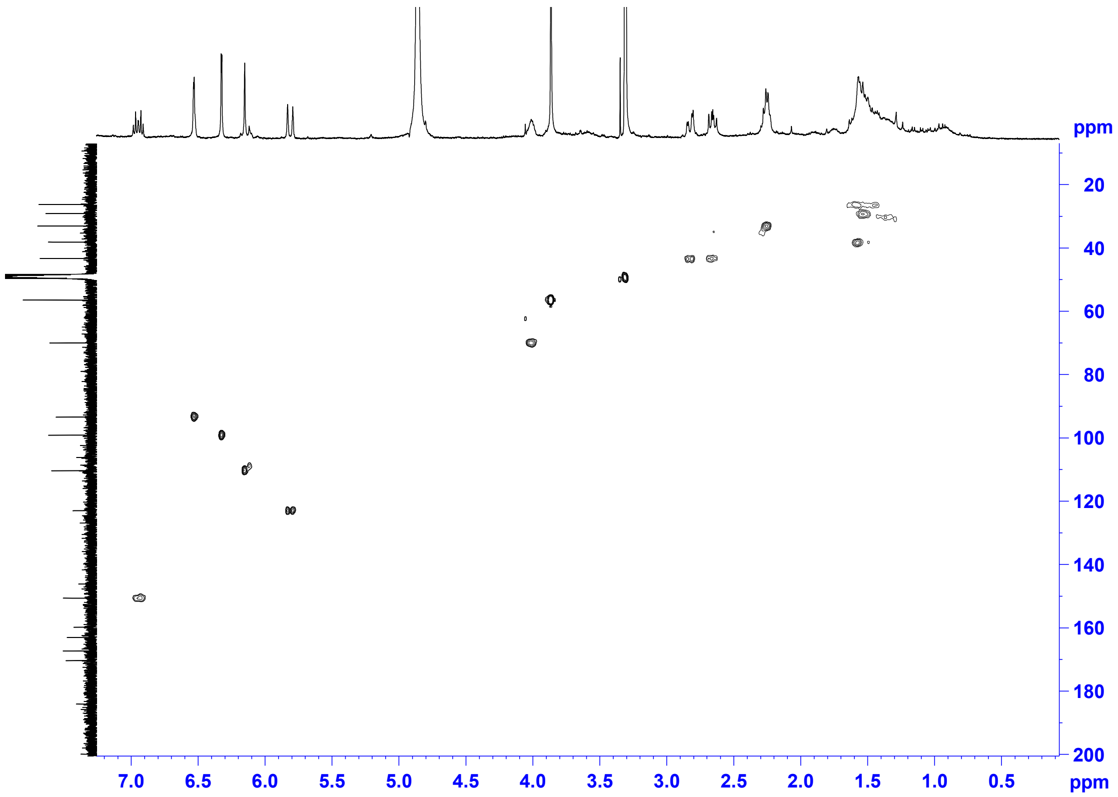

Figure S33. HSQC Spectrum of **6** in Methanol-d_4.

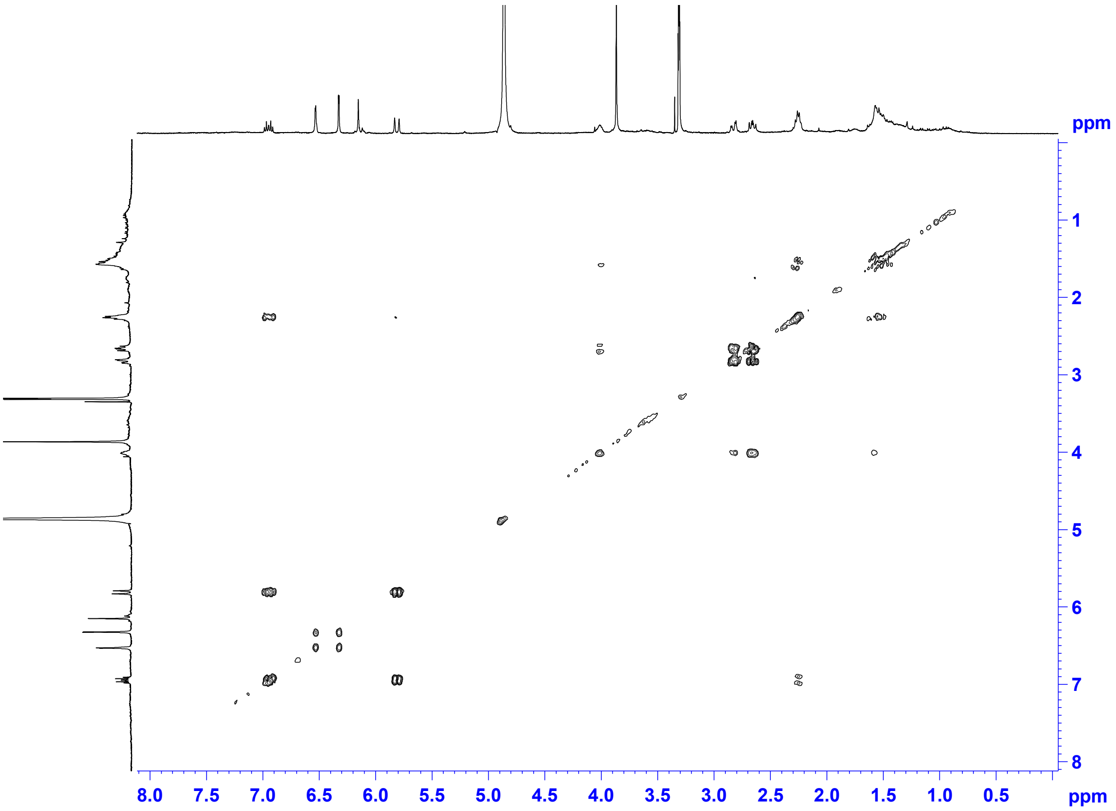

Figure S34. ^{1}H-^{1}H COSY Spectrum of **6** in Methanol-d_4.

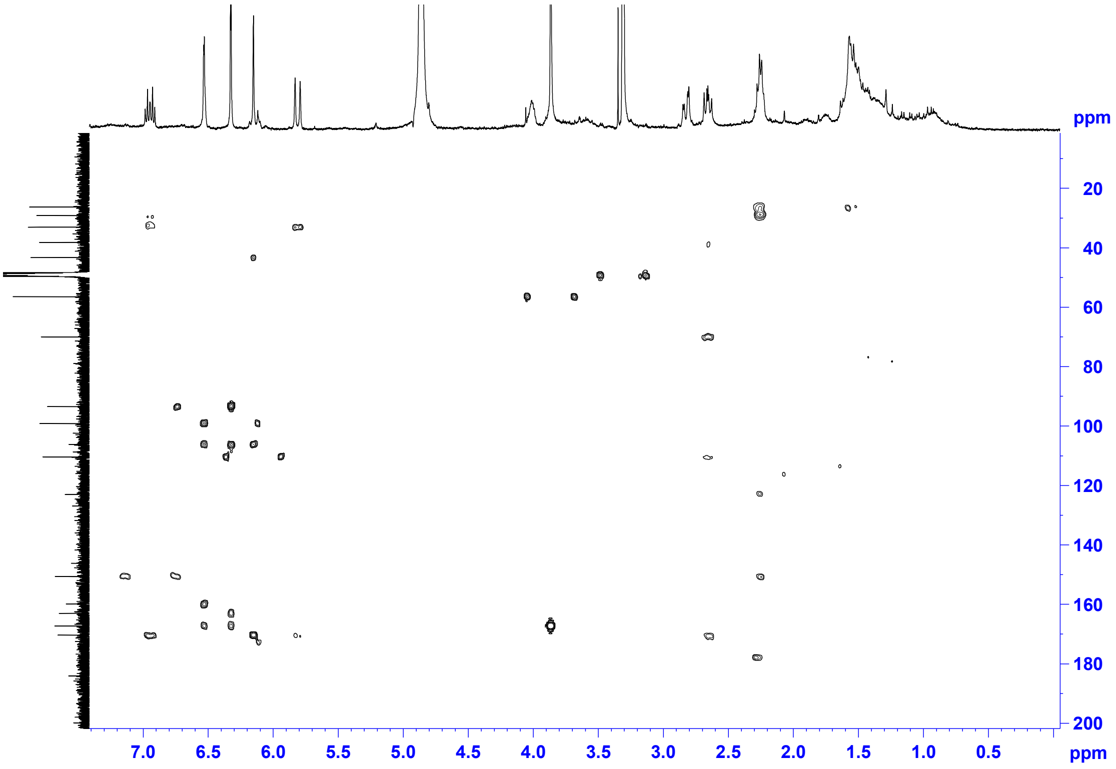

Figure S35. HMBC Spectrum of **6** in Methanol-d_4.

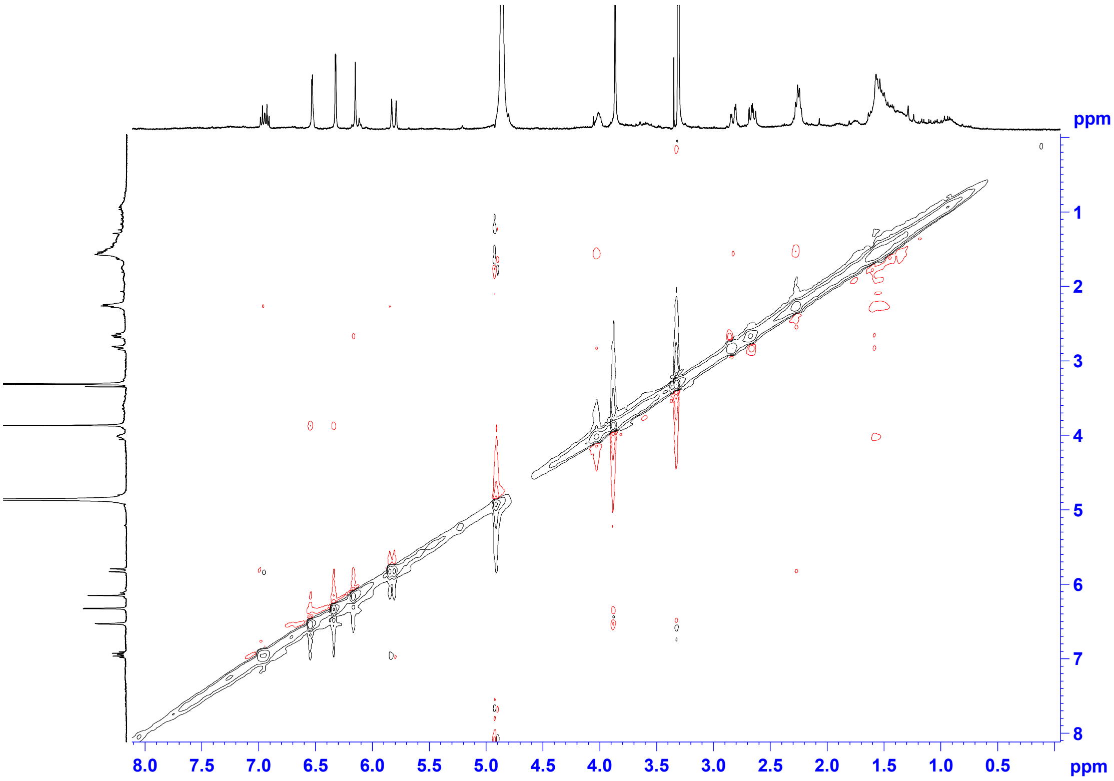

Figure S36. NOESY Spectrum of **6** in Methanol-d_4.

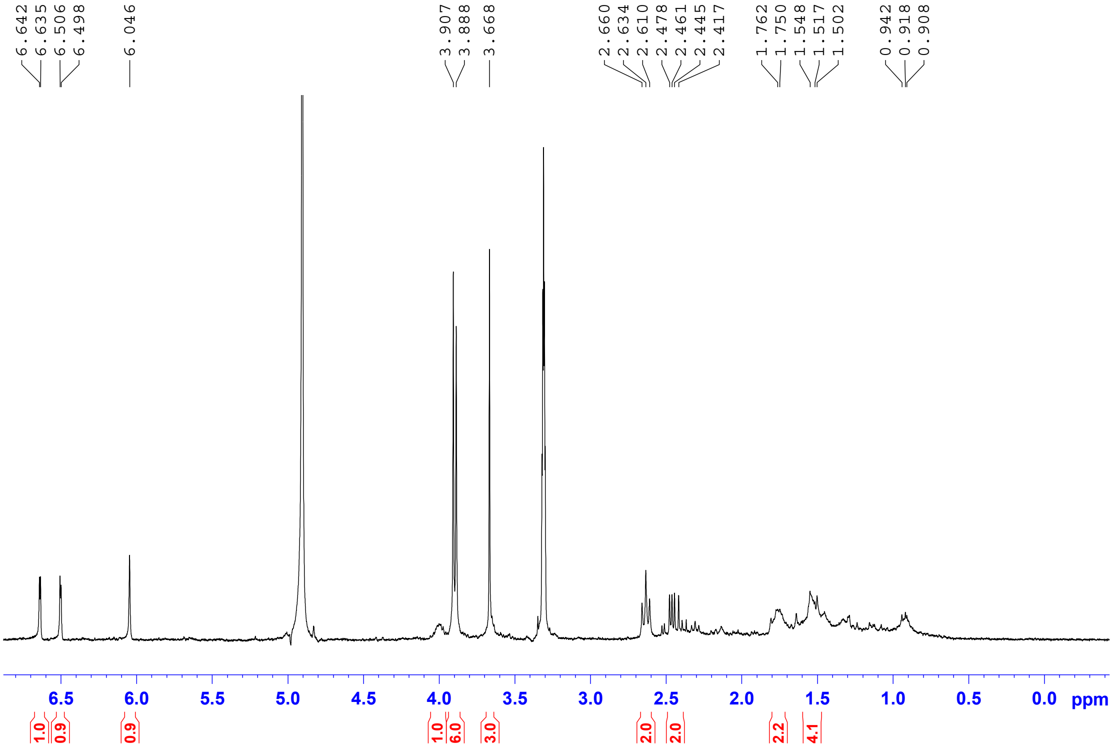

Figure S37. ^{1}H NMR Spectrum of **7** in Methanol-d_4 (400 MHz).

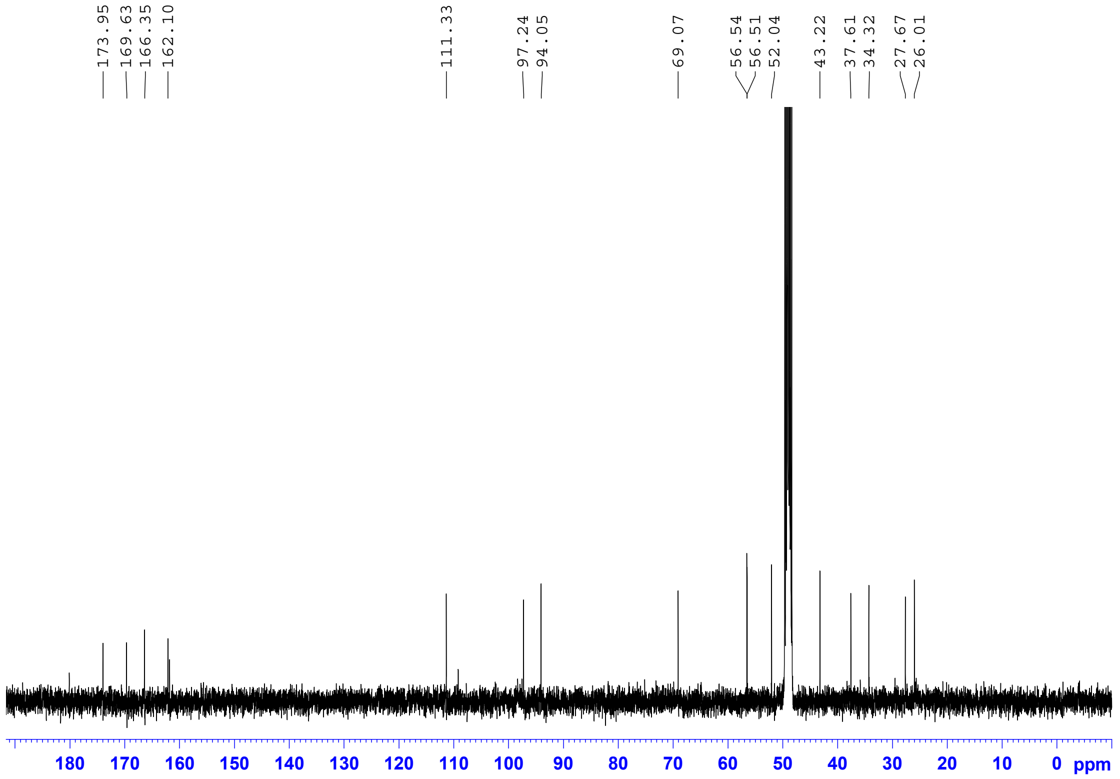

Figure S38. ^{13}C NMR Spectrum of **7** in Methanol-d_4 (100 MHz).

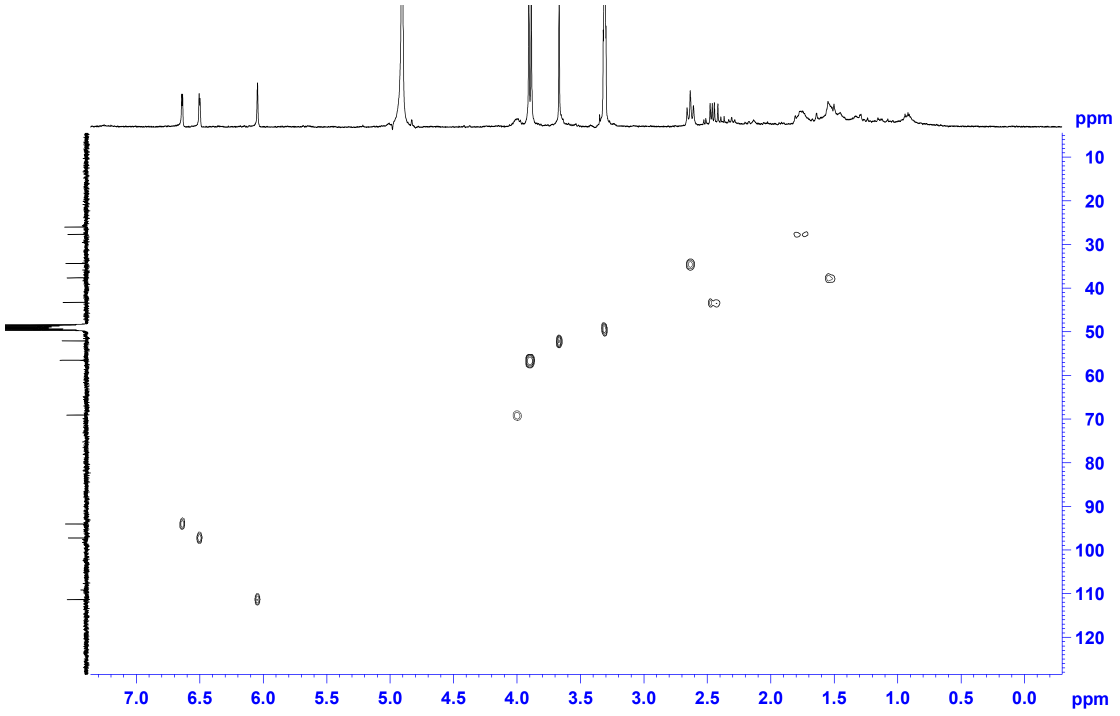

Figure S39. HSQC Spectrum of **7** in Methanol-d_4.

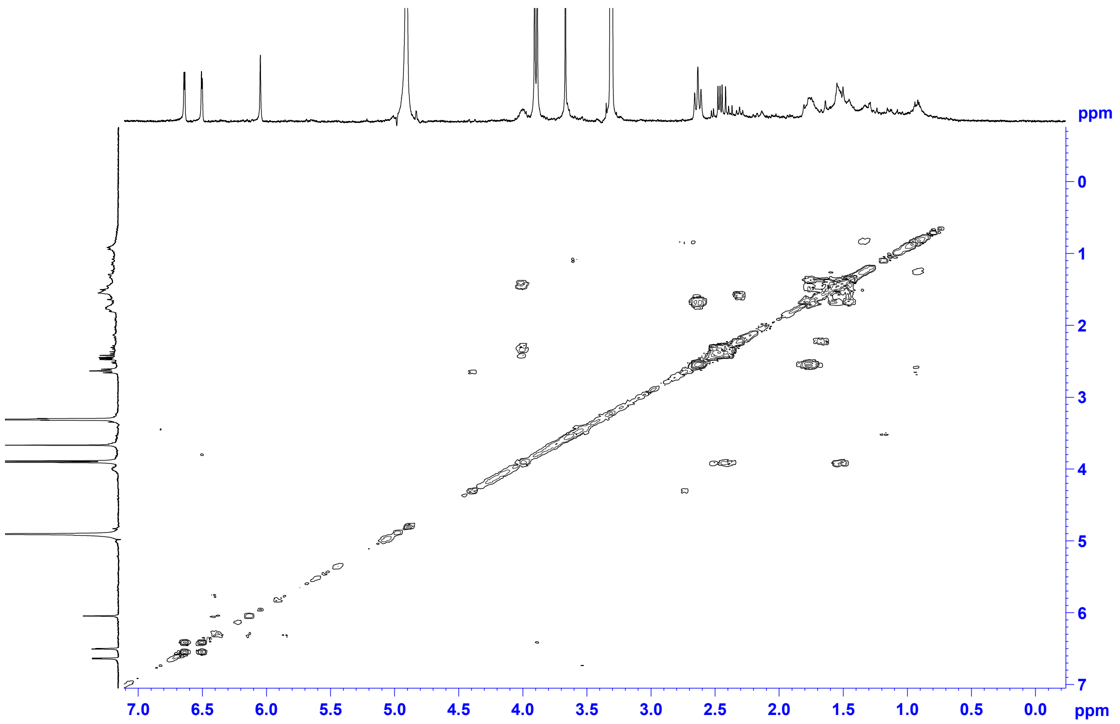

Figure S40. ^1H-^1H COSY Spectrum of **7** in Methanol-*d*4.

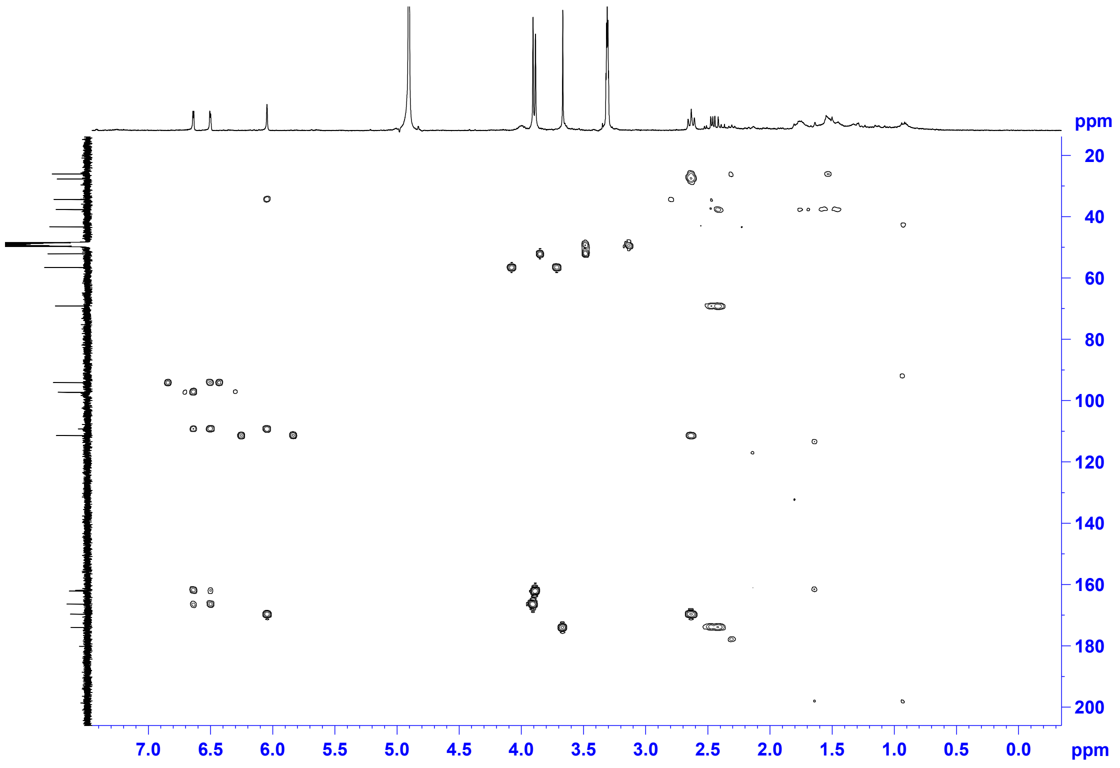

Figure S41. HMBC Spectrum of **7** in Methanol-*d*4.

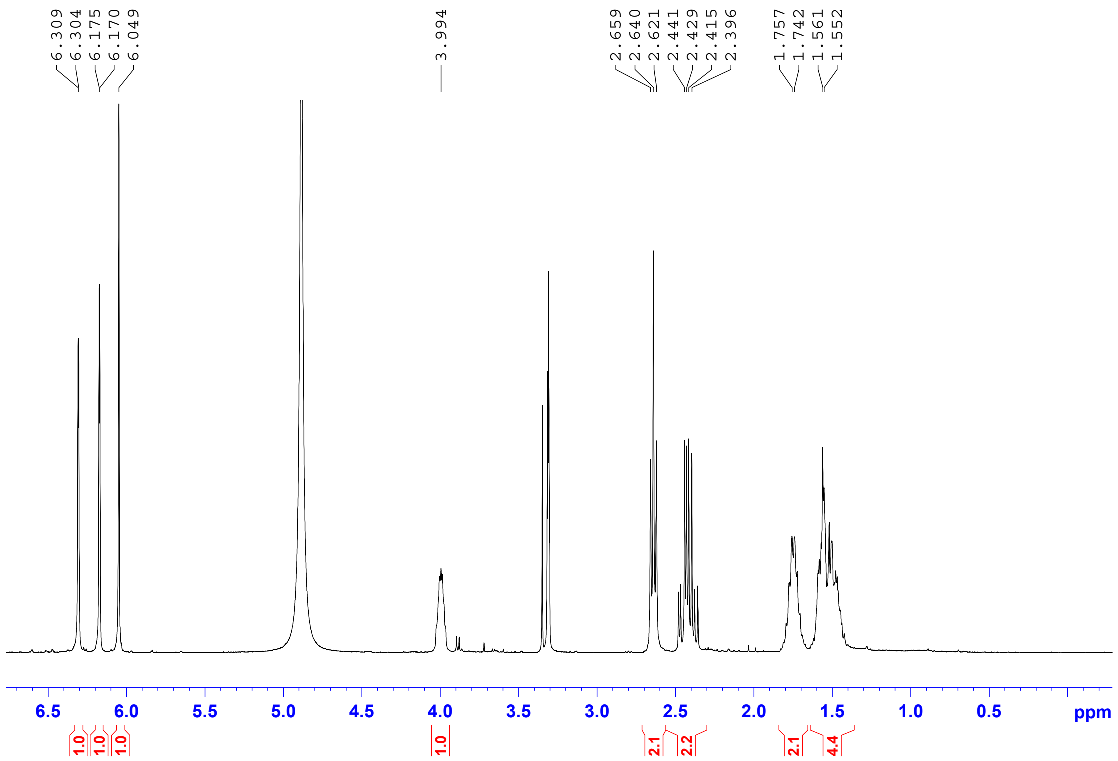

Figure S42. ^{1}H NMR Spectrum of **8** in Methanol-d_4 (400 MHz).

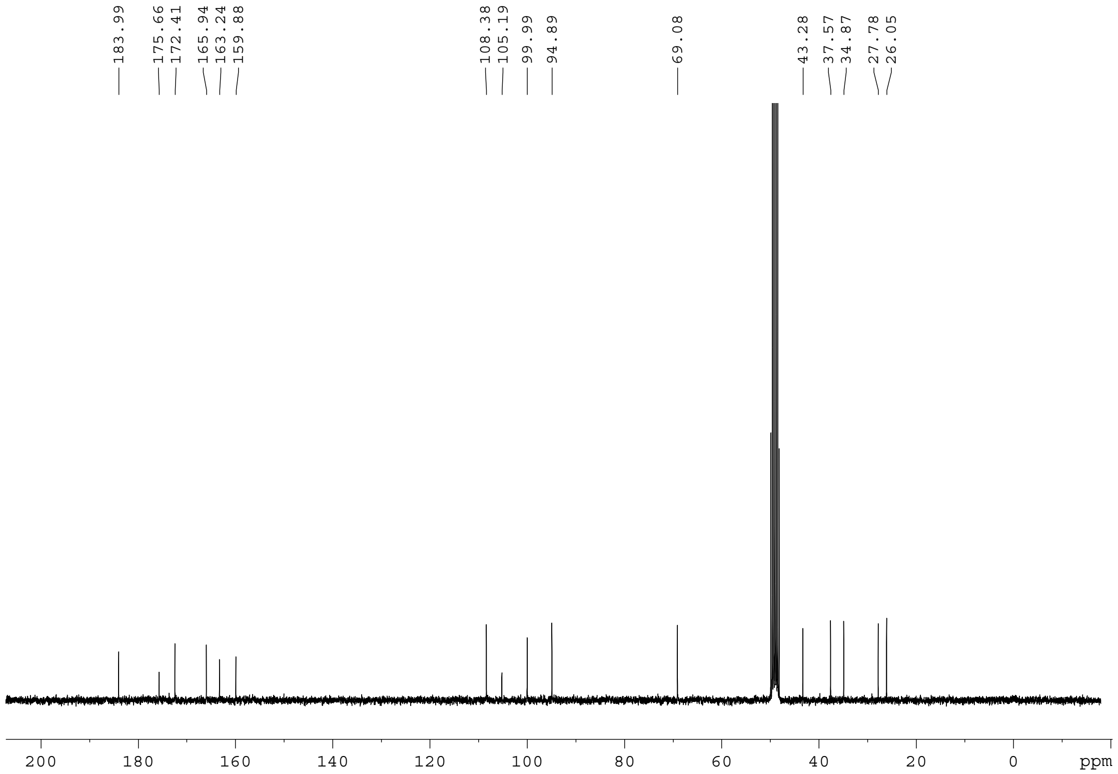

Figure S43. ^{13}C NMR Spectrum of **8** in Methanol-d_4 (100 MHz).

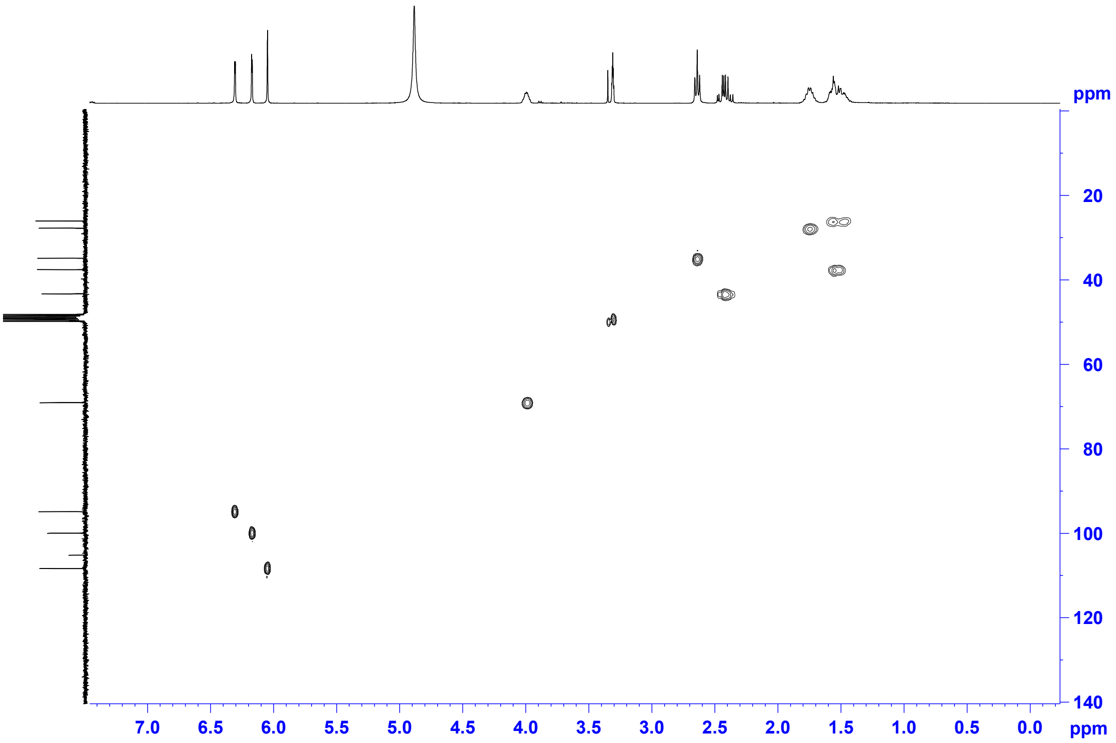

Figure S44. HSQC Spectrum of **8** in Methanol-d_4.

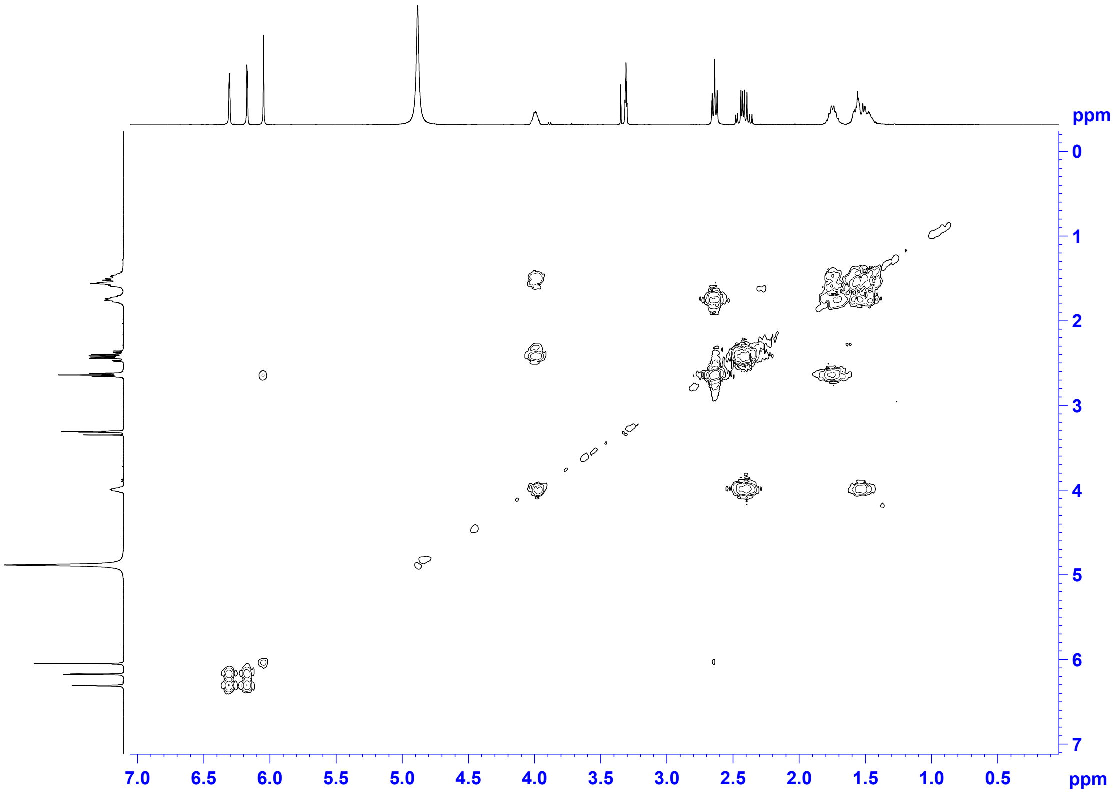

Figure S45. ^{1}H-^{1}H COSY Spectrum of **8** in Methanol-d_4.

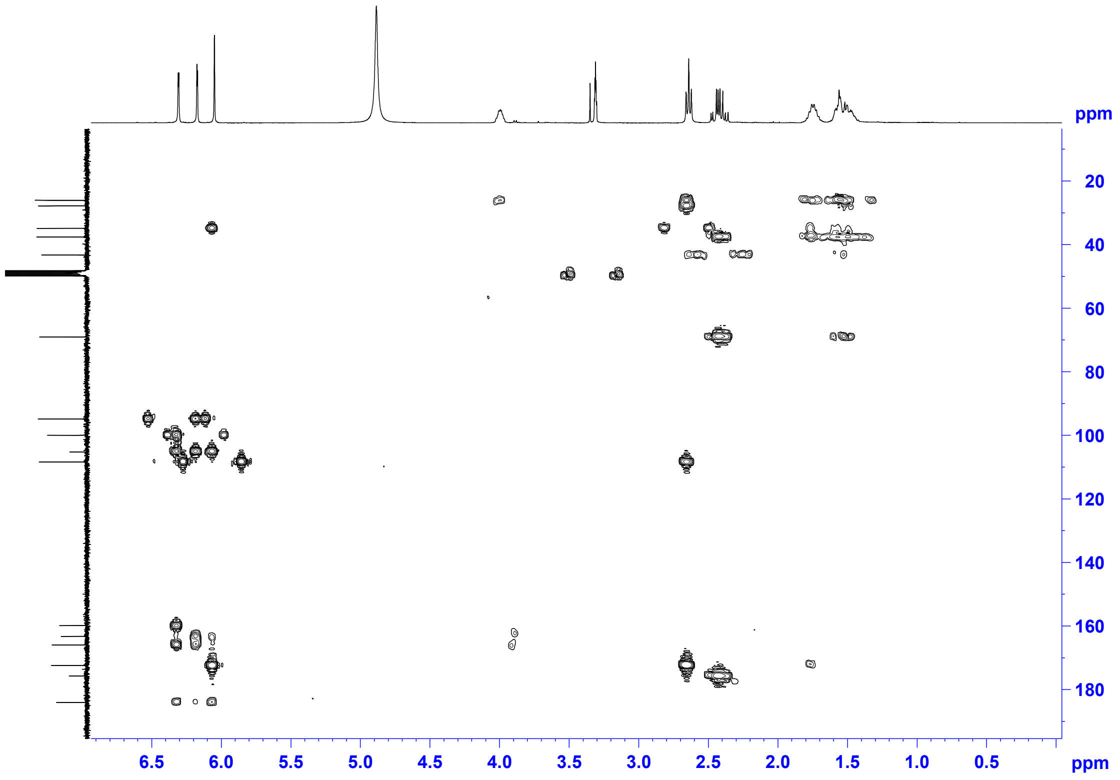

Figure S46. HMBC Spectrum of **8** in Methanol-d_4.

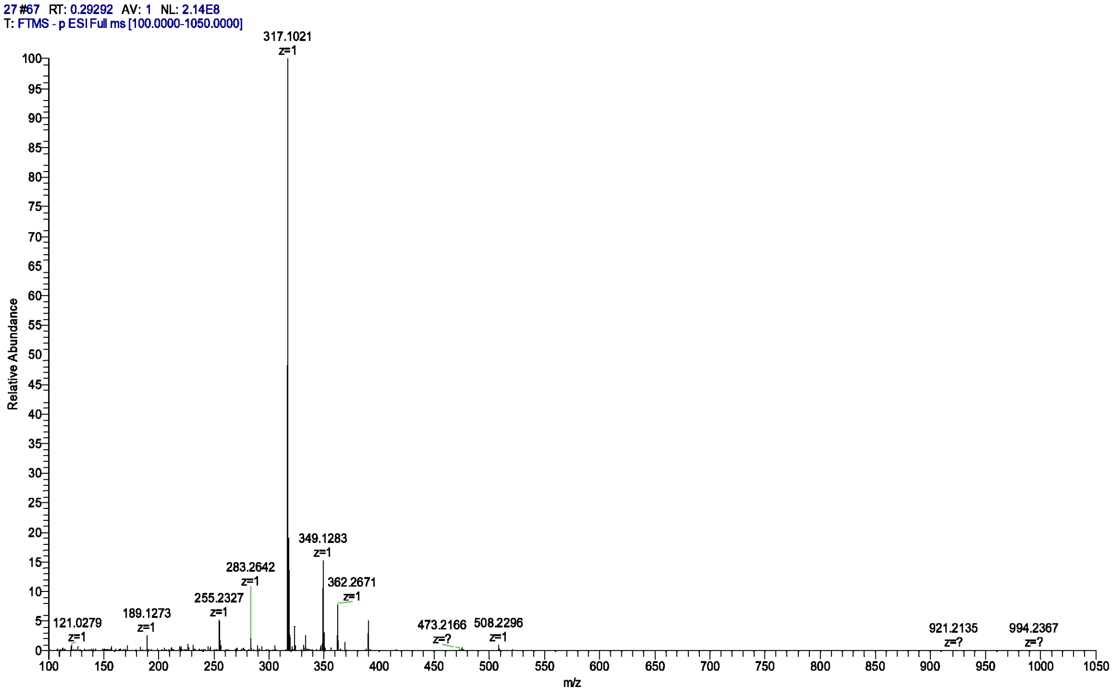

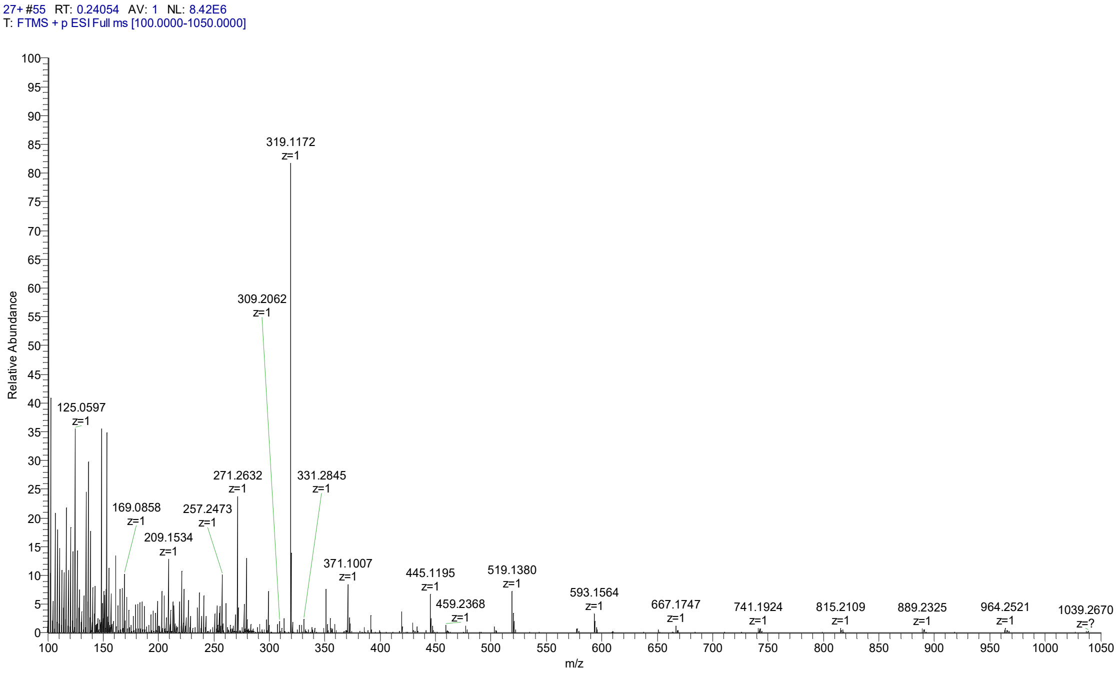

Figure S47. HRESIMS spectrum of **1.**

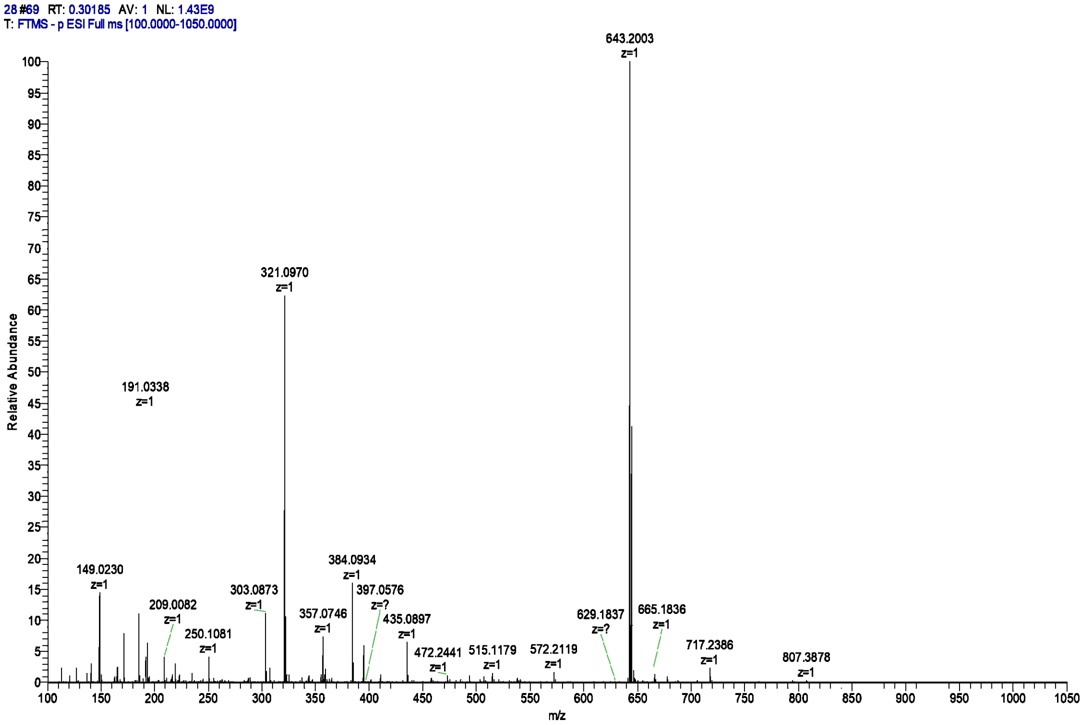

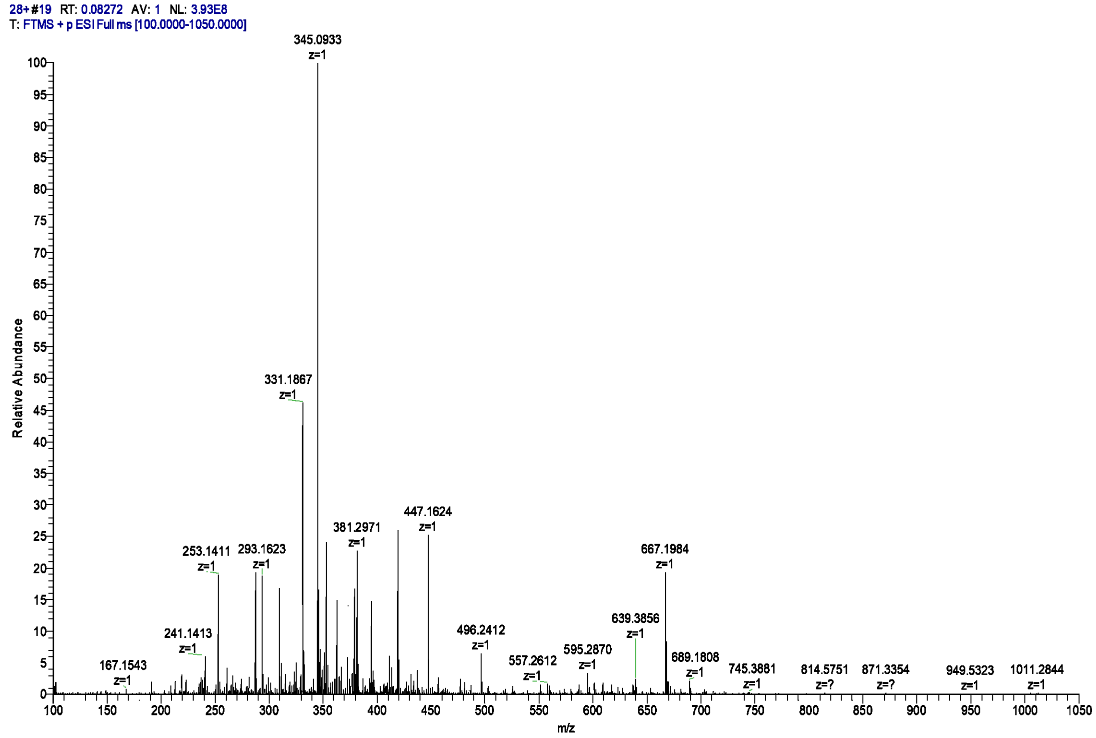

Figure S48. HRESIMS spectrum of **2.**

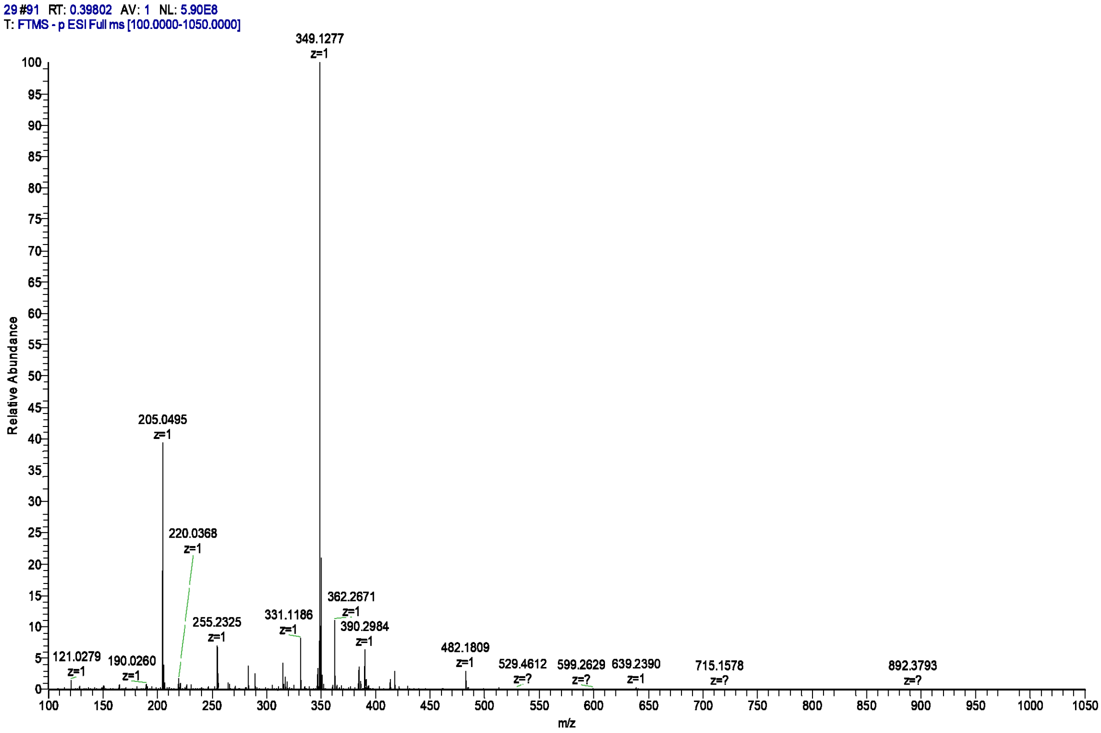

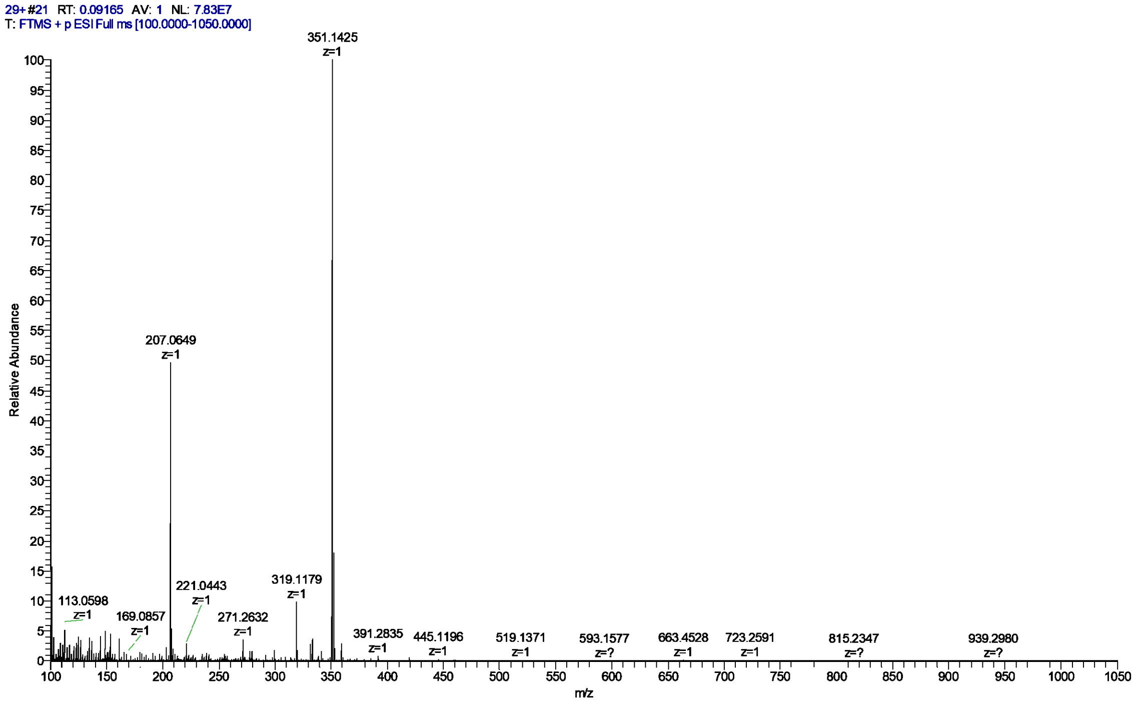

Figure S49. HRESIMS spectrum of **3.**

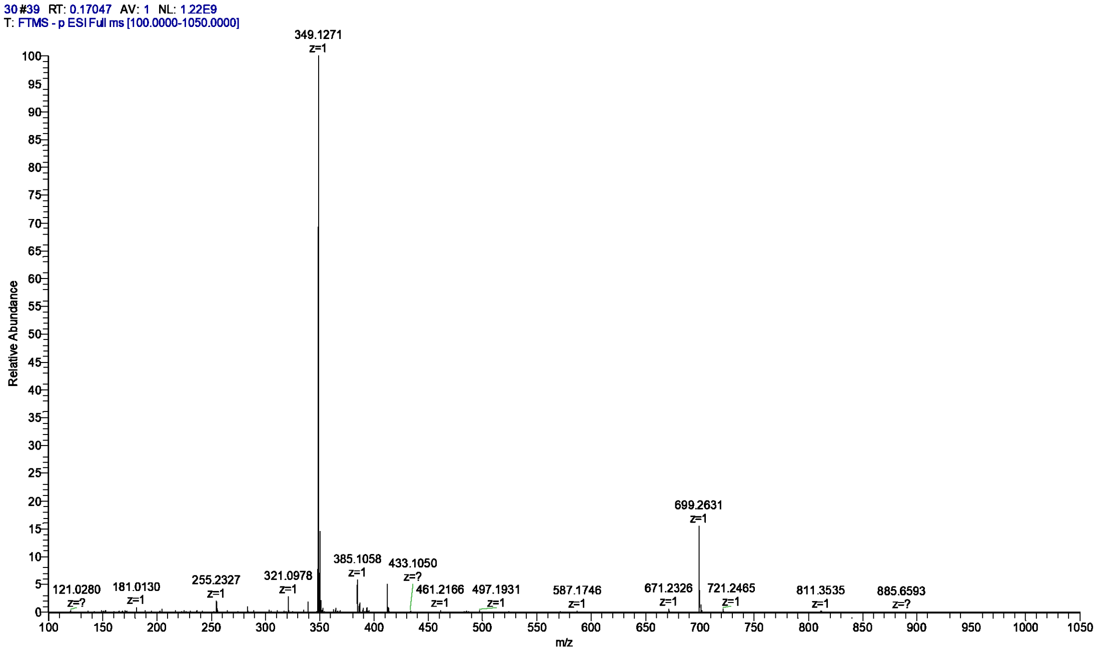

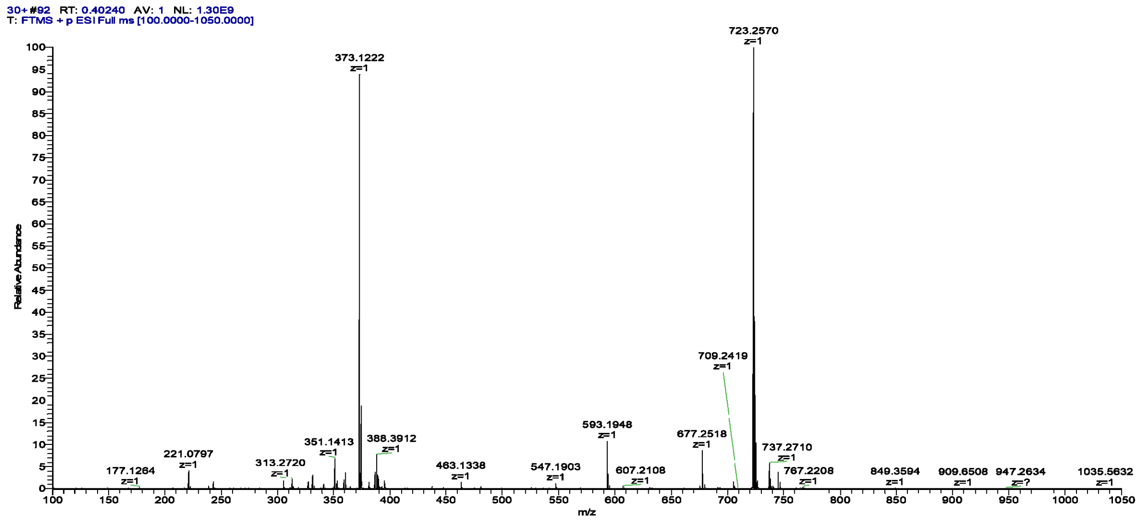

Figure S50. HRESIMS spectrum of **4.**

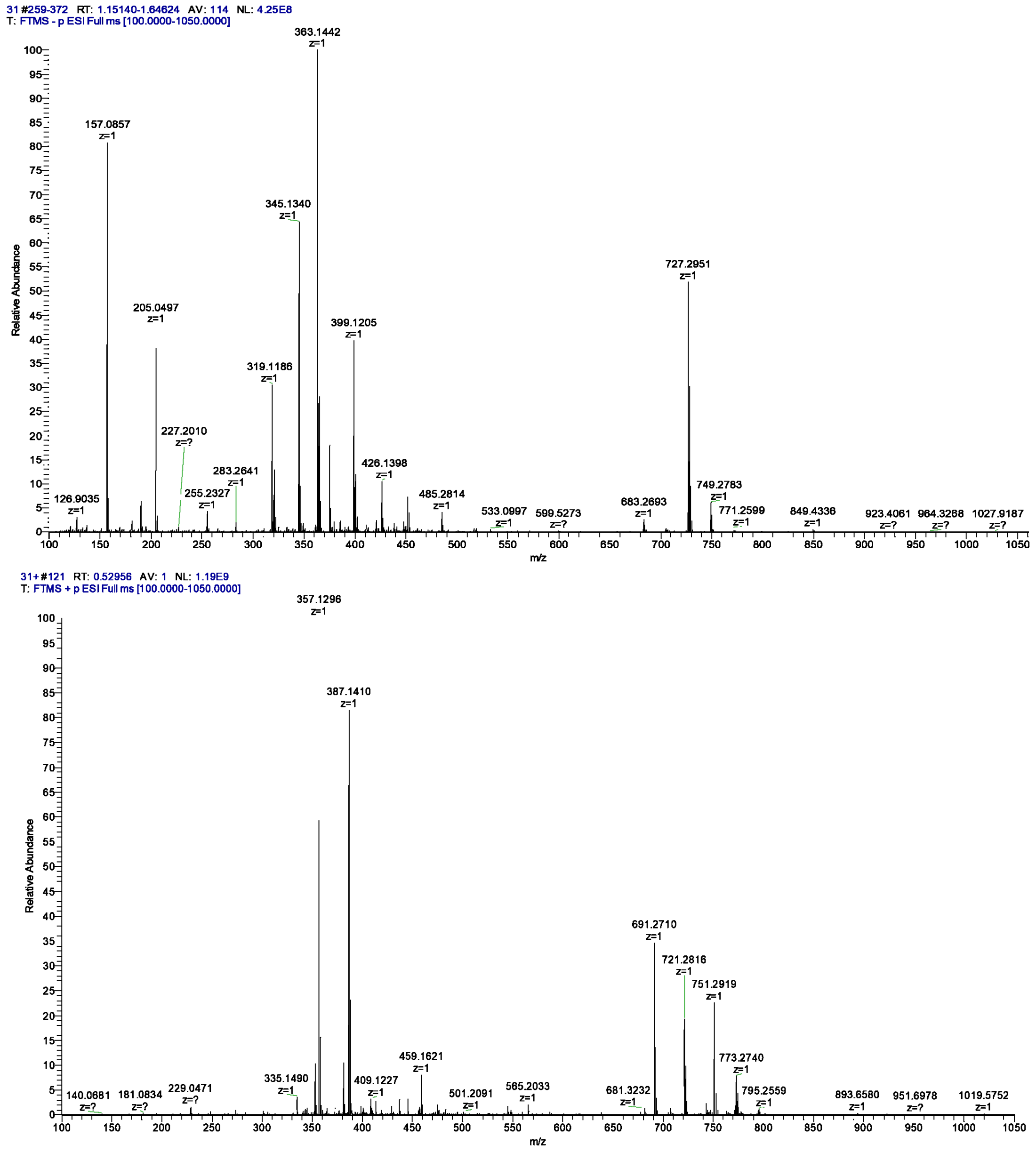

Figure S51. HRESIMS spectrum of **5.**

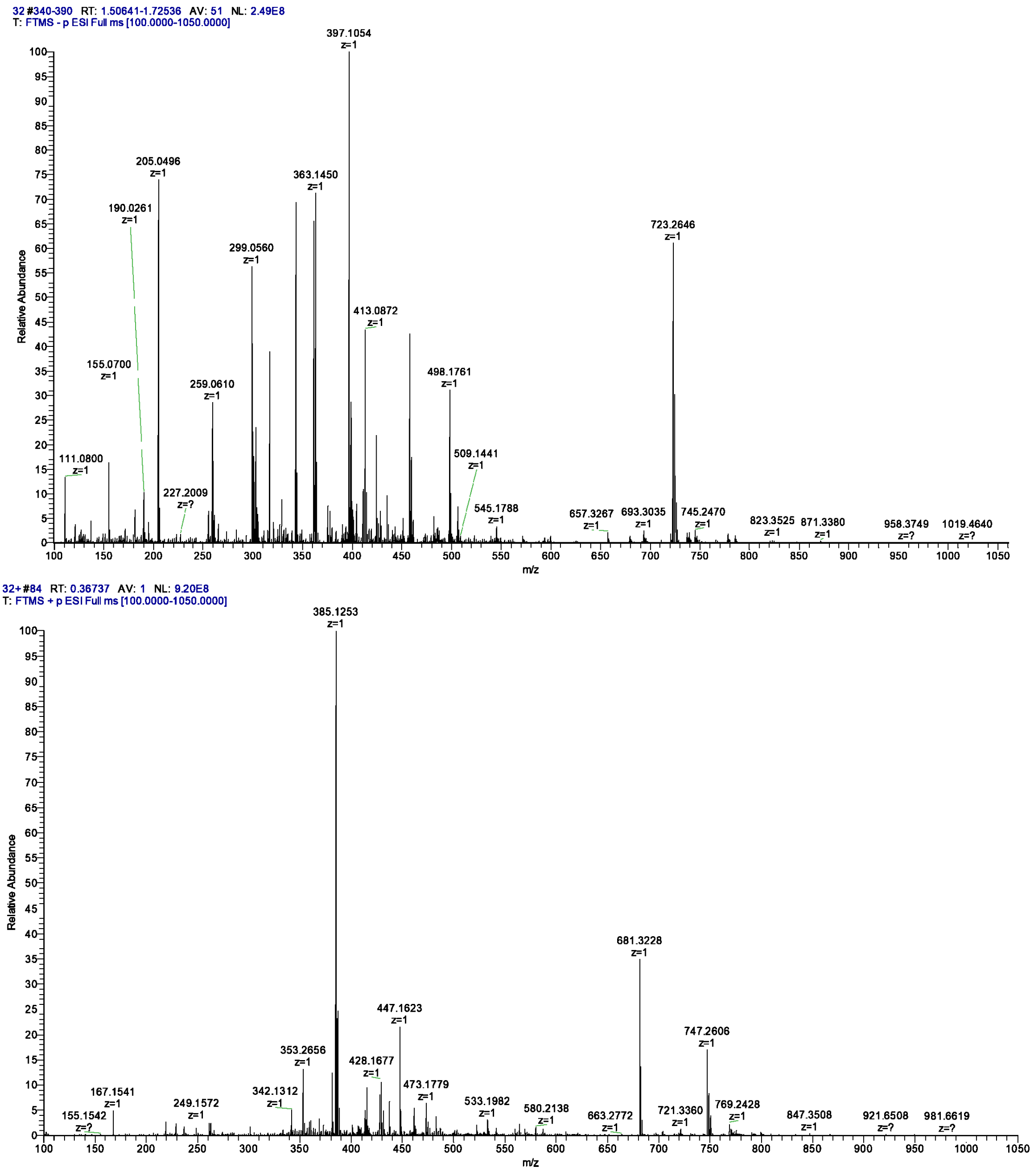

Figure S52. HRESIMS spectrum of **6.**

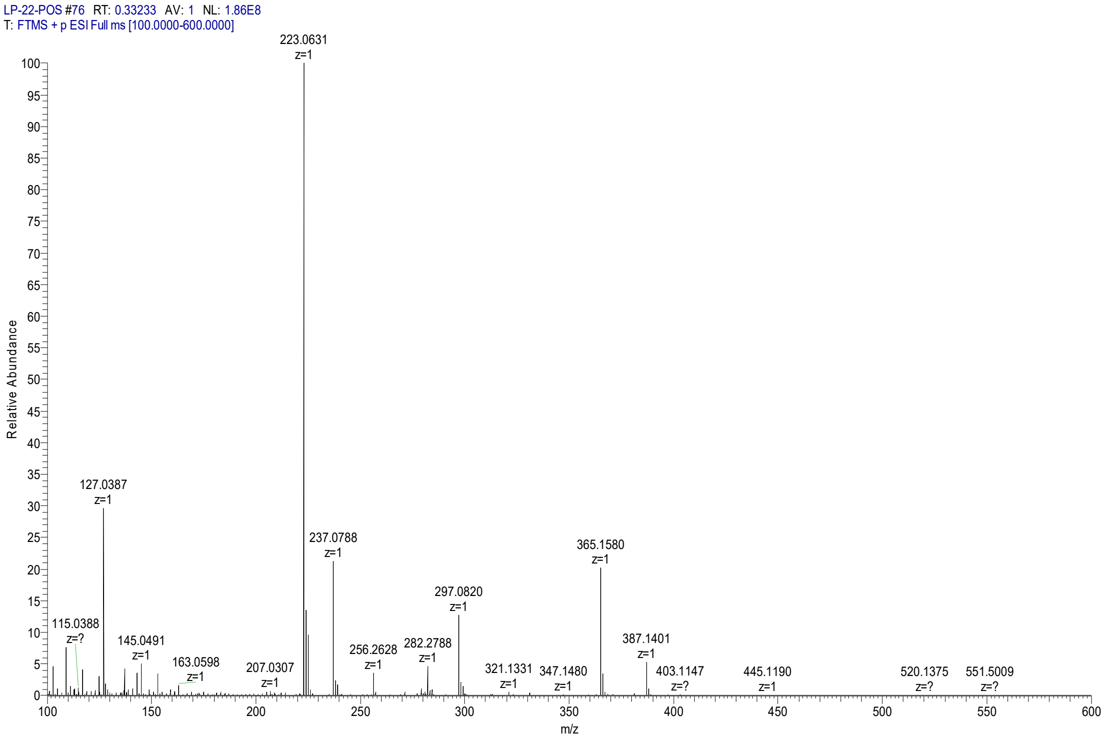

Figure S53. HRESIMS spectrum of **7.**

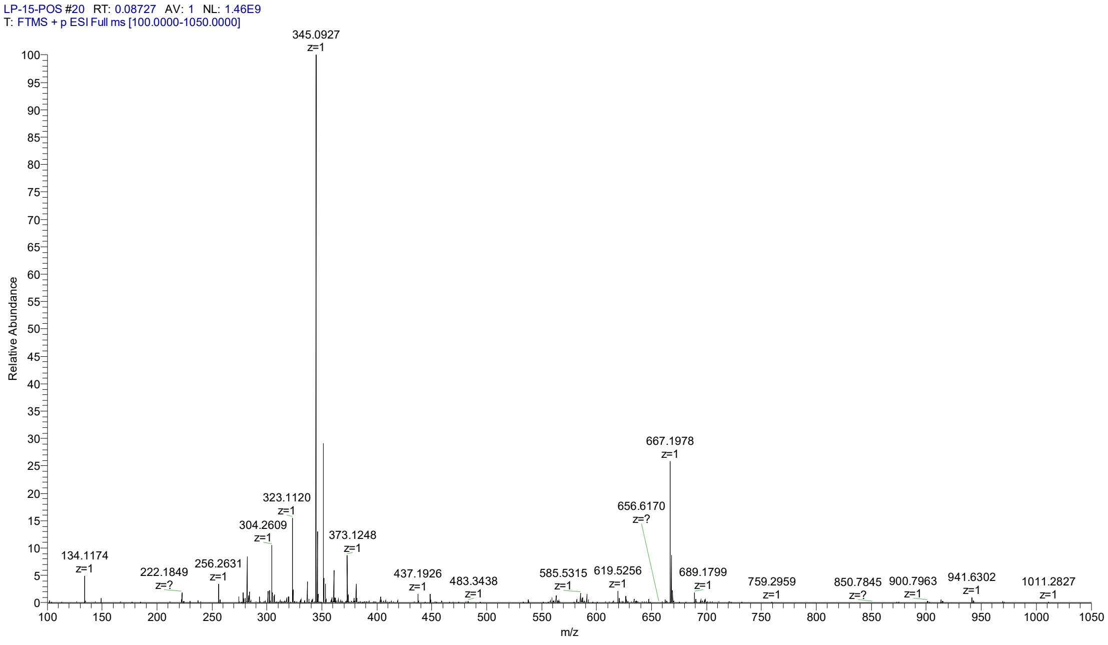

Figure S54. HRESIMS spectrum of **8.**

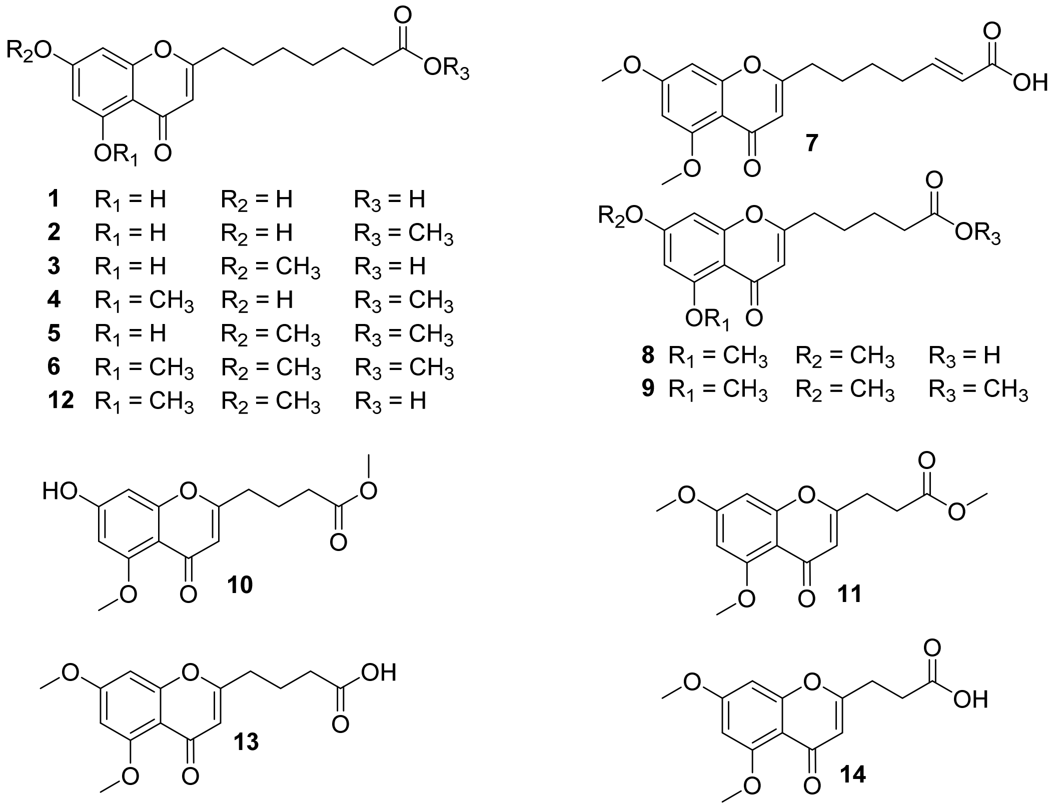

Figure S55. Structures of Reported Analogs (Penithochromones A–L (**1**–**12**), 4-(5,7-dimethoxy-4-oxo-4H-chromen-2-yl)butanoic acid (**13**), and 3-(5,7-dimethoxy-4-oxo-4H-chromen-2-yl)propanoic acid (**14**)).

Table S1. Calculation of the specific rotation of **1**.

Conformer	Gibbs free energy (298.15 K)			Specific rotations	Averaged
	G (Hartree)	ΔE (kcal/mol)	Population (%)		
C1	−1110.118975	0	45.81	−216.43	
C2	−1110.118844	0.000131	39.88	−132.51	−175.60
C3	−1110.117877	0.001098	14.31	−164.32	
